# On-treatment derived neutrophil-to-lymphocyte ratio and survival with palbociclib and endocrine treatment: analysis of a multicenter retrospective cohort and the PALOMA-2/3 study with immune correlates

**DOI:** 10.1186/s13058-022-01601-4

**Published:** 2023-01-12

**Authors:** Chang Gon Kim, Min Hwan Kim, Jee Hung Kim, Seul-Gi Kim, Gun Min Kim, Tae Yeong Kim, Won-Ji Ryu, Jee Ye Kim, Hyung Seok Park, Seho Park, Young Up Cho, Byeong Woo Park, Seung Il Kim, Joon Jeong, Joohyuk Sohn

**Affiliations:** 1 Division of Medical Oncology, Department of Internal Medicine, Yonsei Cancer Center, Yonsei University College of Medicine, 50-1 Yonsei-Ro, Seodaemun-Gu, Seoul, 03722 Republic of Korea; 2 Division of Medical Oncology, Department of Internal Medicine, Gangnam Severance Hospital, Yonsei University College of Medicine, Seoul, Republic of Korea; 3 Institute for Breast Cancer Precision Medicine, Yonsei University College of Medicine, Seoul, Republic of Korea; 4 Avison Biomedical Research Center, Yonsei University College of Medicine, Seoul, Republic of Korea; 5 Division of Breast Surgery, Department of Surgery, Yonsei Cancer Center, Yonsei University College of Medicine, Seoul, Republic of Korea; 6 Division of Breast Surgery, Department of Surgery, Gangnam Severance Hospital, Yonsei University College of Medicine, 712 Eonjuro, Gangnam-Gu, Seoul, 06273 Republic of Korea

**Keywords:** Advanced breast cancer, CDK4/6 inhibitor, Neutrophil-to-lymphocyte ratio, Palbociclib

## Abstract

**Background:**

Cyclin-dependent kinase 4 and 6 (CDK4/6) inhibitors have been established as a standard treatment for hormone receptor (HR)-positive, human epidermal growth factor receptor 2 (HER2)-negative advanced breast cancer (ABC); however, predictive biomarkers with translational relevance have not yet been elucidated.

**Methods:**

Data from postmenopausal women who received the CDK4/6 inhibitor palbociclib and letrozole for HR-positive, HER2-negative ABC from tertiary referral centers were analyzed (*N* = 221; exploratory cohort). Pre- and on-treatment neutrophil-to-lymphocyte ratio (NLR) and derived NLR (dNLR; neutrophil/[leukocyte-neutrophil]) were correlated with survival outcomes. Data from the PALOMA-2 (NCT01740427) and PALOMA-3 studies (NCT01942135) involving patients treated with endocrine treatment with or without palbociclib were also analyzed (validation cohort). Prospectively enrolled patients (*N* = 20) were subjected to immunophenotyping with circulating immune cells to explore the biological implications of immune cell dynamics.

**Results:**

In the exploratory cohort, palbociclib administration significantly reduced leukocyte, neutrophil, and lymphocyte counts on day 1 of cycle 2. Although the baseline dNLR was not significantly associated with progression-free survival (PFS), higher on-treatment dNLRs were associated with worse PFS (hazard ratio = 3.337, *P* < 0.001). In the PALOMA-2 validation cohort, higher on-treatment dNLRs were associated with inferior PFS in patients treated with palbociclib and letrozole (hazard ratio = 1.498, *P* = 0.009), and reduction in the dNLR after treatment was predictive of a survival benefit (hazard ratio = 1.555, *P* = 0.026). On-treatment dNLRs were also predictive of PFS following palbociclib and fulvestrant treatment in the PALOMA-3 validation cohort. Using flow cytometry analysis, we found that the CDK4/6 inhibitor prevented T cell exhaustion and diminished myeloid-derived suppressor cell frequency.

**Conclusions:**

On-treatment dNLR significantly predicted PFS in patients with HR-positive, HER2-negative ABC receiving palbociclib and endocrine treatment. Additionally, we observed putative systemic immune responses elicited by palbociclib, suggesting immunologic changes upon CDK4/6 inhibitor treatment.

**Supplementary Information:**

The online version contains supplementary material available at 10.1186/s13058-022-01601-4.

## Background

The cyclin D-cyclin-dependent kinases 4 and 6 (CDK4/6)-retinoblastoma (RB) pathway is a fundamental component of cell cycle regulation and is implicated in the initiation and progression of various malignancies [1, 2]. In particular, alterations in the cyclin D-CDK4/6-Rb pathway contribute to both de novo and acquired resistance to endocrine treatment in patients with hormone receptor (HR)-positive breast cancer [3, 4]. Hence, this pathway is an important target for pharmacologic permutations to overcome endocrine resistance. Concordantly, CDK4/6 inhibitors exhibit robust and durable clinical activity in treating HR-positive, human epidermal growth factor receptor 2 (HER2)-negative advanced breast cancer (ABC) [5–8]. Among CDK4/6 inhibitors, palbociclib demonstrates significant efficacy in combination with letrozole as first-line therapy (PALOMA-2) or fulvestrant as later-line therapy (PALOMA-3) in HR-positive, HER2-negative ABC [9–12]. Although phase III studies consistently show clinical benefits of CDK4/6 inhibitors irrespective of menopausal status, visceral metastasis, or ethnicity, a subset of patients treated with CDK4/6 inhibitors exhibit an early progression, warranting the identification of biomarkers to predict treatment outcome. Unfortunately, previously identified potential biomarkers are inconsistent, have limited clinical feasibility, and do not reflect the dynamic changes induced by CDK4/6 inhibitors [13, 14].

In addition to the essential role of CDK4/6 in cell cycle progression through RB phosphorylation, which underlies the proliferation of HR-positive breast cancer cells [15], novel functions have been discovered [16]. CDK6 is involved in the proliferation and differentiation of hematopoietic cells [17–19]. Consequently, hematologic adverse events, including neutropenia, commonly occur after CDK4/6 inhibitor administration [9, 20]. Moreover, CDK4/6 inhibitors enhance antitumor immunity through tumor infiltration and activation of T cells via transcriptional reprogramming [21, 22]. Combining immune checkpoint and CDK4/6 inhibitors synergistically delays tumor growth, providing a rationale for combination strategies comprising CDK4/6 inhibitors and immunotherapies. In this context, a series of clinical trials testing various combinations of CDK4/6 and immune checkpoint inhibitors are currently underway.

Baseline and/or treatment-related lymphopenia is associated with poor clinical outcomes in patients with various malignancies, including metastatic breast cancer [23–25]. Meanwhile, neutrophilia is generally associated with poor outcomes [26], consistent with preclinical evidence that phenotypic and functional characteristics of neutrophils primarily overlap with those of polymorphonuclear myeloid-derived suppressor cells in cancer [27, 28]. Accordingly, the neutrophil-to-lymphocyte ratio (NLR) is commonly used as an inflammatory biomarker in cancer patients, and a higher NLR is commonly associated with poor prognosis in patients with breast cancer [29].

It is well known that CDK4/6 inhibitors modulate the absolute numbers and composition of circulating immune cells [30]; however, their predictive value has not been determined. We hypothesized that the dynamic changes in the immune cell populations induced by CDK4/6 inhibitors could reflect the resulting antitumor response and may be predictive of patient survival. To test this hypothesis, we analyzed a multicenter retrospective cohort of palbociclib-treated patients with HR-positive, HER2-negative ABC as well as patients of prospective phase III studies (PALOMA-2 and PALOMA-3). The composition and characteristics of immune cells in the peripheral blood of patients being treated with CDK4/6 inhibitors were evaluated using flow cytometry analysis of T cell and myeloid-derived suppressor cell (MDSC) markers to garner further mechanistic insights.

## Patients and methods

### Exploratory cohort

Patients from the metastatic breast cancer database registry of two tertiary referral centers (161 patients from Yonsei Cancer Center and 60 from Gangnam Severance Hospital) in the Republic of Korea who received palbociclib and letrozole as first-line treatment for HR-positive, HER2-negative ABC between January 2017 and April 2020 constituted the exploratory cohort. The exploratory cohort included premenopausal and postmenopausal patients. All premenopausal patients underwent bilateral salpingo-oophorectomy before palbociclib and letrozole treatment because the national health insurance policy did not allow the use of luteinizing hormone-releasing hormone agonists with palbociclib and letrozole in premenopausal patients. Baseline patient demographics were collected, including age, initial stage, recurrence type, disease-free interval, metastatic site and number, treatment outcome, and survival data. This study was approved by the institutional review board of each institution (IRB number: 4-2016-0574), and informed consent was obtained from all patients.

### Validation cohort

Results from the exploratory cohort were validated using the double-blind multicenter phase III PALOMA-2 study (NCT01740427) [9]. In this study, women with HR-positive, HER2-negative ABC were randomly assigned to receive 2.5 mg letrozole orally continuously in combination with either palbociclib (125 mg/day orally for 3 weeks followed by 1 week without drug administration in a 4-week cycle) or a matching placebo. Postmenopausal women with no prior systemic therapy for advanced disease, an Eastern Cooperative Oncology Group (ECOG) performance status of 0–2, and adequate organ function were eligible for enrollment. The primary study end-point was investigator-assessed progression-free survival (PFS), defined as the time from randomization to radiologically confirmed disease progression or death during the study. Women with an adequate follow-up period (> 29 days) and available leukocyte and neutrophil counts at cycle 2 day 1 with a window period of 7 days were analyzed (*N* = 619; 410 patients from the palbociclib- and letrozole-treated groups and 209 patients from the placebo and letrozole-treated groups). Lymphocyte counts were not collected in the PALOMA-2 study case report. Patients treated with palbociclib (125 mg/day orally for 3 weeks followed by 1 week without drug administration in a 4-week cycle) and fulvestrant (500 mg intramuscularly every 2 weeks for the first three injections and then every 4 weeks) in the PALOMA-3 study (NCT01942135) were also analyzed [12]. Both PALOMA-2 and PALOMA-3 study data were obtained from Pfizer at the investigators’ request through the Vivli platform (https://vivli.org).

### Peripheral immune cell counts

Leukocyte, neutrophil, and lymphocyte counts in peripheral blood were collected at baseline, cycle 1 day 15, and cycle 2 day 1 of treatment. The NLR was calculated as neutrophil count/lymphocyte count. The derived NLR (dNLR) was calculated as neutrophil count/(leukocyte count—neutrophil count) and was used as an NLR surrogate because the lymphocyte count was unavailable in the PALOMA-2 and PALOMA-3 validation cohort.

### Next-generation sequencing (NGS)

Tumor samples were collected and targeted DNA and RNA sequencing were performed using TruSight Tumor 500 (Illumina, San Diego, CA, USA). DNA and RNA from formalin-fixed paraffin-embedded (FFPE) tissue using a Qiagen All Prep DNA/RNA FFPE kit (Qiagen, Hilden, Germany). Library preparation was performed according to the manufacturer’s instructions. After hybridization capture-based target enrichment, pair-ended sequencing (2 × 150 bp) was performed using a NextSeq sequencer (Illumina) according to the manufacturer’s instructions as previously described [31].

### Blood-based immune monitoring with flow cytometry

We prospectively collected peripheral blood at baseline, cycle 1 day 15, and cycle 2 day 1 in an independent prospective cohort of 20 patients treated with CDK4/6 inhibitors for ABC from September 2020 to April 2021 at the Yonsei Cancer Center. Immune cells were isolated as previously described [32]. Single-cell suspensions were incubated with the LIVE/DEAD Fixable Near-IR Dead Cell Stain kit (Thermo Fisher Scientific) and then stained with fluorochrome-conjugated antibodies for 15 min at room temperature. For intracellular staining, cells were permeabilized and fixed with the FoxP3 staining buffer kit (Thermo Fisher Scientific) and further stained with fluorochrome-conjugated antibodies. Flow cytometry analysis was conducted using an LSR Fortessa system (BD Biosciences) and FlowJo software version 10.4.0 (Tree Star Inc.). To characterize CD8^+^ T cell exhaustion, the relative frequency of PD-1^+^ cells and mean fluorescent intensities of TOX, NFATc1, and CTLA-4 in PD-1^+^CD8^+^ T cells were measured based on previously published articles [33, 34]. Relative ratios of MDSCs and T cells, numbers of monocytic MDSCs (M-MDSC; CD14^+^CD11b^+^CD15^−^HLA-DR^−^CD3^−^CD19^−^ cells), or polymorphonuclear MDSCs (PMN-MDSC; CD15^+^CD11b^+^CD14^−^HLA^−^DR^−^CD3^−^CD19^−^ cells) were obtained by dividing those of T cells (CD3^+^CD19^−^CD14^−^ cells) based on previously defined gating strategies [35]. This study was approved by the institutional review board of Severance Hospital (IRB number: 4–2020-0472). Informed consent was obtained from patients. The reagents used for flow cytometry are described in Additional file 1: Table S1.

### Statistical analysis

Statistical analyses were performed using SPSS version 25 (IBM), R version 3.5.3. (R Foundation for Statistical Computing), and GraphPad Prism (GraphPad Software). Chi-squared and Mann–Whitney U tests were used to compare patient characteristics between two groups. Paired *t*-tests were used for pair-wise comparisons before and after treatment. Mean values with standard deviations or ranges are presented for continuous variables. Correlations between variables were analyzed based on a linear regression model. The log-rank maximization method was used to find the best dNLR cut-off values for PFS in the exploratory cohort, the PALOMA-2 validation cohort treated with letrozole plus placebo, and the PALOMA-3 exploratory cohort using the MaxStat R package. Clinical benefit was defined as complete response, partial response, or stable disease lasting more than 24 weeks. PFS was defined as the time interval from the initiation of palbociclib treatment to radiologically confirmed disease progression or patient death, and overall survival (OS) was defined as the time interval from the initiation of palbociclib treatment to patient death in the exploratory cohort. Patient survival was monitored until November 3, 2020. Survival differences among the patient groups were compared using log-rank tests and depicted using Kaplan–Meier plots. A Cox proportional hazards regression model was used to estimate the hazard ratio, and multivariate analysis was conducted to adjust variables associated with patient outcomes. In the PALOMA-2 validation cohort, multiple log-rank tests for PFS comparing letrozole plus palbociclib therapy with letrozole monotherapy were conducted in palbociclib-treated patients with increasing dNLR cut-offs to investigate the correlation between palbociclib-driven PFS benefit and on-treatment dNLR.

## Results

### Patient characteristics of the exploratory cohort

A total of 233 postmenopausal women who received palbociclib and letrozole as first-line treatment for HR-positive, HER2-negative ABC from two tertiary referral centers were analyzed. Patients who did not receive the second cycle of treatment (*N* = 8) or lacked complete blood cell count data at baseline or cycle 2 day 1 (*N* = 4) were excluded, resulting in 221 patients being included in the final analysis (Table 1). The median age was 55 years, and most patients were of Asian ethnicity. Visceral and bone-only metastases were documented in 123 (55.7%) and 44 (19.9%) patients, respectively. After a median follow-up of 20.7 months, the median PFS was 31.5 months, and the median OS was not reached.

**Table 1:** Baseline characteristics of the exploratory and validation cohorts treated with letrozole and palbociclib

Characteristics	Validation cohort	Exploratory cohort	*P* value
	(*N* = 410)	(*N* = 221)	
Age			< 0.001
Median	62 (30–89)	55 (27–87)	
< 65 years old	248 (60.5%)	174 (78.7%)	
≥ 65 years old	162 (39.5%)	47 (21.3%)	
< 55 years old	98 (23.9%)	103 (46.6%)	
≥ 55 years old	312 (76.1%)	118 (53.4%)	
Race			< 0.001
White	320 (78%)	4 (1.8%)	
Asian	59 (14.4%)	216 (97.7%)	
Black	7 (1.7%)	0 (0.0%)	
Other	24 (5.9%)	1 (0.5%)	
Initial stage			0.002
I	48 (11.7%)	31 (14.0%)	
II	124 (30.2%)	66 (29.9%)	
III	69 (16.8%)	26 (11.8%)	
IV	127 (31.0%)	91 (41.2%)	
N/A	42 (10.2%)	7 (3.2%)	
Recurrence type			0.034
Locoregional	9 (2.2%)	5 (2.3%)	
Distant	274 (66.8%)	125 (56.6%)	
Newly diagnosed	127 (31.0%)	91 (41.2%)	
Disease-free interval			0.120
Newly metastatic disease	155 (37.8%)	102 (46.2%)	
≤ 12 month	90 (22.0%)	44 (19.9%)	
> 12 month	165 (40.2%)	75 (33.9%)	
Disease site			0.135
Visceral	197 (48.0%)	123 (55.7%)	
Nonvisceral	213 (52.0%)	98 (44.3%)	
Bone-only	96 (23.4%)	44 (19.9%)	
Number of disease sites			0.826
1	129 (31.5%)	70 (31.7%)	
2	106 (25.9%)	64 (29.0%)	
3	101 (24.6%)	50 (22.6%)	
≥ 4	74 (18.0%)	37 (16.7%)	

N/A, not assessed

### Dynamic immune cell count changes after palbociclib and letrozole treatments

Because hematologic adverse events are frequently observed following palbociclib administration [36], we analyzed dynamic changes in immune cell counts before and after treatment. Absolute numbers of leukocytes, neutrophils, and lymphocytes decreased from 6.20 ± 0.14 × 10^3^/mm^3^, 3.84 ± 0.11 × 10^3^/mm^3^, and 1.74 ± 0.05 × 10^3^/mm^3^ to 2.85 ± 0.09 × 10^3^/mm^3^, 1.06 ± 0.06 × 10^3^/mm^3^, and 1.42 ± 0.04 × 10^3^/mm^3^, respectively (Additional file 2: Figure S1A-S1C; *P* < 0.001). The fold change of each cell population was calculated to compensate for differences between patients, and these showed similar trends (Additional file 2: Figure S1D-S1F; *P* < 0.001). The fold change in the number of neutrophils (0.30 ± 0.02) was more pronounced than that of lymphocytes (0.87 ± 0.02) after treatment (Additional file 2: Figure S1G), indicating that palbociclib had distinct effects on different immune cell populations. Accordingly, the NLR was significantly reduced after palbociclib administration (Additional file 2: Figure S1H-S1I; *P* < 0.001).

### Predictive value of baseline and on-treatment immune cell counts for survival outcomes

**Fig. 1:**
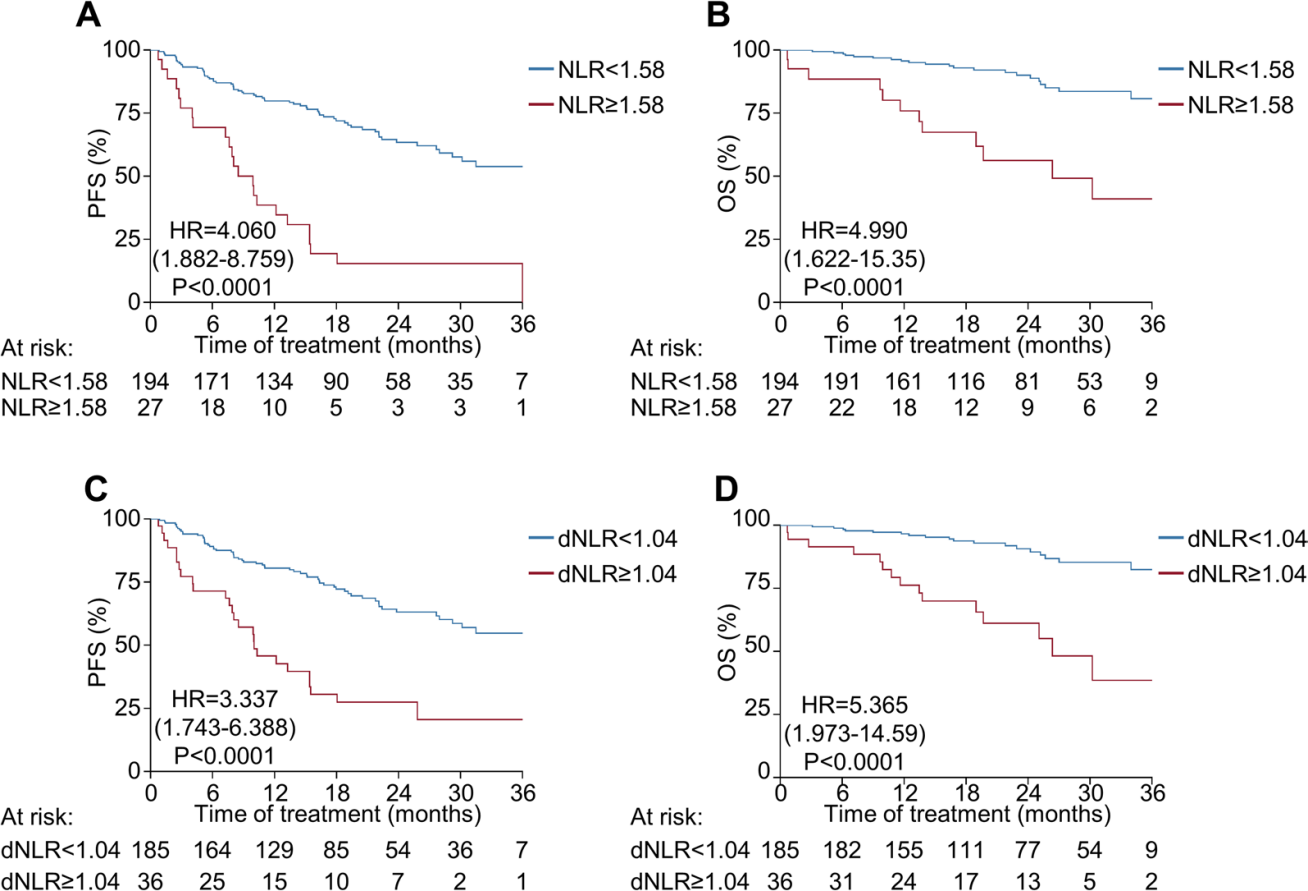
Survival outcomes according to on-treatment neutrophil-to-lymphocyte ratios (NLRs) and derived NLRs in exploratory cohort (*N* = 221). **A**, **B** Progression-free survival (**A**) and overall survival (**B**) according to the neutrophil-to-lymphocyte ratio (cut-off = 1.58) after treatment (cycle 2 day 1). **C**, **D** Progression-free survival (**C**) and overall survival (**D**) according to the derived neutrophil-to-lymphocyte ratio (cut-off = 1.04) after treatment (cycle 2 day 1). dNLR, derived neutrophil-to-lymphocyte ratio; HR, hazard ratio; NLR, neutrophil-to-lymphocyte ratio; OS, overall survival; PFS, progression-free survival

Next, we investigated whether baseline and on-treatment immune cell counts were associated with survival outcomes. Increased lymphocyte counts at baseline were associated with favorable survival outcomes (hazard ratio = 0.689 for PFS per 1000/mm^3^ increments of lymphocyte count, *P* = 0.031). However, leukocyte and neutrophil counts at baseline were not significantly associated with PFS (Additional file 1: Table S2). Variables measured at cycle 1 day 15 were not significantly correlated with survival, suggesting that they are not informative for the following analysis. Notably, increased neutrophil counts at cycle 2 day 1 were associated with inferior PFS (hazard ratio = 1.453 for PFS per 1000/mm^3^ increments of the neutrophil count, *P* < 0.001), whereas increased lymphocyte counts at cycle 2 day 1 were associated with superior PFS (hazard ratio = 0.636 for PFS per 1000/mm^3^ increments of lymphocyte count, *P* = 0.013). Correspondingly, an increased NLR on cycle 2 day 1 was significantly associated with worse PFS (hazard ratio = 1.641 for PFS per unit increment of NLR, *P* < 0.001). In contrast, baseline NLR marginally predicted PFS (hazard ratio = 1.115, *P* = 0.069). When baseline and on-treatment NLRs were computed, a weak positive correlation was observed (Additional file 3: Figure S2A). Bivariate analysis with baseline and on-treatment NLRs revealed that on-treatment NLRs independently predicted PFS (hazard ratio = 1.625, *P* < 0.001). In contrast, baseline NLRs did not significantly predict patient outcome (hazard ratio = 1.070, *P* = 0.284, Additional file 1: Table S3). When a maximized log-rank test was conducted, an NLR cut-off value of 1.58 reasonably predicted PFS (hazard ratio = 4.060 for NLR ≥ 1.58 [*N* = 27] vs. NLR < 1.58 [*N* = 194], *P* < 0.001, Fig. 1A). A similar result was obtained when the analysis was extended to OS (hazard ratio = 4.990 for NLR ≥ 1.58 vs. NLR < 1.58, *P* < 0.001, Fig. 1B). Taken together, these data support the predictive potential of on-treatment NLRs after palbociclib treatment.

### Interchangeability of NLR and dNLR

**Fig. 2:**
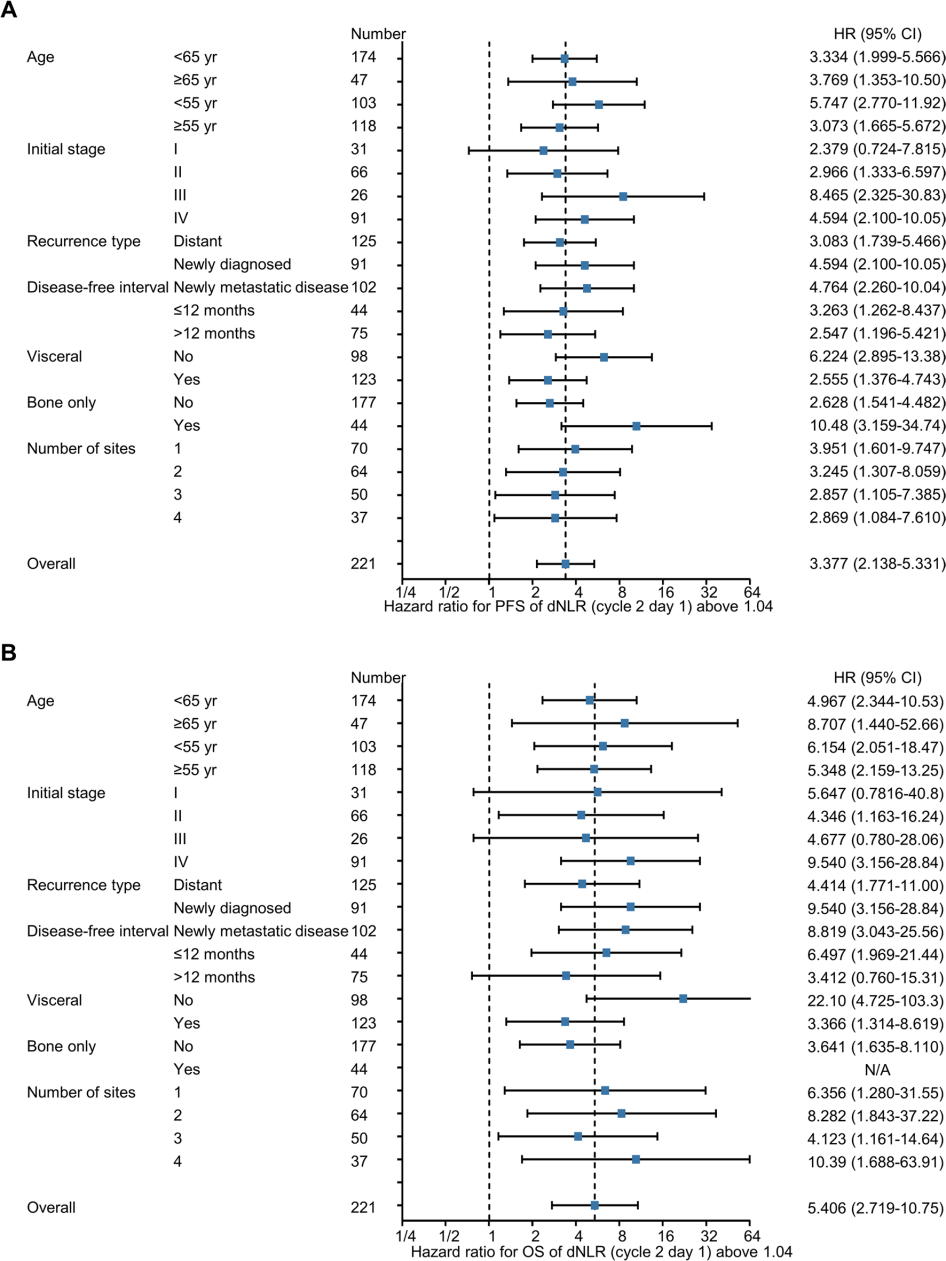
Forest plot of the derived neutrophil-to-lymphocyte ratio on survival outcomes (*N* = 221). **A**, **B** Forest plot of the dNLR on progression-free survival (**A**) and overall survival (**B**) in the exploratory cohort. CI, confidence interval; dNLR, derived neutrophil-to-lymphocyte ratio; HR, hazard ratio; OS, overall survival; PFS, progression-free survival

As the NLR requires both neutrophil and lymphocyte counts, the general application of this biomarker is difficult when lymphocyte counts are unavailable. Thus, we introduced an index called the dNLR that only requires leukocyte and neutrophil counts [37]. As observed with the NLR, the dNLR was significantly reduced after treatment (Additional file 4: Figure S3A-S3B). In addition, the NLR and dNLR showed a strong correlation both at baseline and on-treatment (Additional file 4: Figure S3C-S3D). Next, we analyzed whether the dNLR can predict survival outcomes by conducting a univariate analysis for PFS. An increased dNLR on cycle 2 day 1 was significantly associated with worse PFS (hazard ratio = 2.524 for PFS per unit increments of dNLR, *P* < 0.001, Additional file 1: Table S2). However, the baseline dNLR marginally predicted PFS (hazard ratio = 1.210, *P* = 0.092), reflecting a weak positive correlation between baseline and on-treatment dNLRs (Additional file 3: Figure S2B). Consistent with the NLR analysis, bivariate analysis of baseline and on-treatment dNLRs also confirmed the independent predictive value of on-treatment dNLRs (Additional file 1: Table S4). Based on maximized log-rank tests, a dNLR cut-off value of 1.04 significantly predicted PFS (hazard ratio = 3.337 for dNLR ≥ 1.04 [*N* = 36] vs. dNLR < 1.04 [*N* = 184], *P* < 0.001, Fig. 1C) and clinical benefit (Additional file 1: Table S5). OS analysis revealed similar results (hazard ratio = 5.365 for dNLR ≥ 1.04 vs. dNLR < 1.04, *P* < 0.001, Fig. 1D), suggesting that the dNLR can replace the NLR as a predictive marker in the absence of lymphocyte count data. There were no differences in baseline characteristics between patients with on-treatment dNLRs above and below 1.04 (Additional file 1: Table S6), suggesting that the dNLR is not influenced by previously known prognostic markers, such as visceral metastasis and tumor burden. In addition, correlative analysis of patients with available next-generation sequencing results (*N* = *26*, Additional file 5: Figure S4A) revealed that specific genetic alteration was not associated with on-treatment dNLR value (Additional file 1: Table S7), although MYC amplification was independently associated with worse PFS (Additional file 1: Table S8). Accordingly, a higher dNLR at cycle 2 day 1 was consistently associated with poor survival outcomes in the various subgroups of patients (Fig. 2A, B) and was predictive of poor PFS (Table 2) and OS (Additional file 1: Table S9) as per both univariate and multivariate analyses. Intriguingly, association between on-treatment dNLR and PFS was also demonstrated in patients treated with ribociclib and letrozole (Additional file 6: Figure S5A-S5C).

**Table 2:** Univariate and multivariate analyses of progression-free survival in the exploratory cohort

Index	Univariate analysis		Multivariate analysis	
	HR (95% CI)	*P* value	HR (95% CI)	*P* value
Age				
≥ 65 years old	0.746 (0.433–1.285)	0.290		
< 65 years old	Reference			
Race				
White	Reference			
Others	0.786 (0.109–5.662)	0.811		
Initial stage				
III–IV	0.785 (0.515–1.197)	0.261		
Others	Reference			
Recurrence type				
Newly diagnosed	0.664 (0.425–1.038)	0.072		
Others	Reference			
Disease-free interval				
Newly metastatic disease	0.625 (0.404–0.966)	0.035	0.574 (0.367–0.898)	0.015
Others	Reference		Reference	
Disease site				
Visceral	1.771 (1.129–2.778)	0.013	1.847 (1.167–2.925)	0.009
Nonvisceral	Reference		Reference	
Bone-only metastasis	0.683 (0.385–1.211)	0.192		
Others	Reference			
Number of disease sites				
≥ 3	1.609 (1.055–2.455)	0.027	1.579 (1.017–2.450)	0.042
1, 2	Reference		Reference	
Derived neutrophil-to-lymphocyte ratio^a^				
≥ 1.04	3.369 (2.134–5.320)	< 0.001	3.314 (2.090–5.256)	< 0.001
< 1.04	Reference		Reference	

CI, confidence interval; HR, hazard ratio

^a^Measured at cycle 2 day 1

### Prospective validation of the predictive value of dNLRs in the PALOMA-2 study

Next, we validated our findings using the PALOMA-2 study [9]. Baseline characteristics were different between the exploratory and validation cohorts with regard to the initial stage, race, and disease status (Table 1), demonstrating the distinct epidemiologic characteristics of ABC according to ethnicity [38]. Letrozole monotherapy did not change leukocyte or neutrophil counts, thus yielding no significant alteration in the dNLR after treatment (Additional file 7: Figure S6A-S6C). Conversely, palbociclib combined with letrozole significantly decreased leukocyte and neutrophil counts (Additional file 7: Figure S6D-S6E). The dNLR value also significantly decreased with palbociclib treatment (Additional file 7: Figure S6F). Consistent with results from the exploratory cohort, patient characteristics did not differ according to the on-treatment dNLR value (Additional file 1: Table S10).

**Fig. 3:**
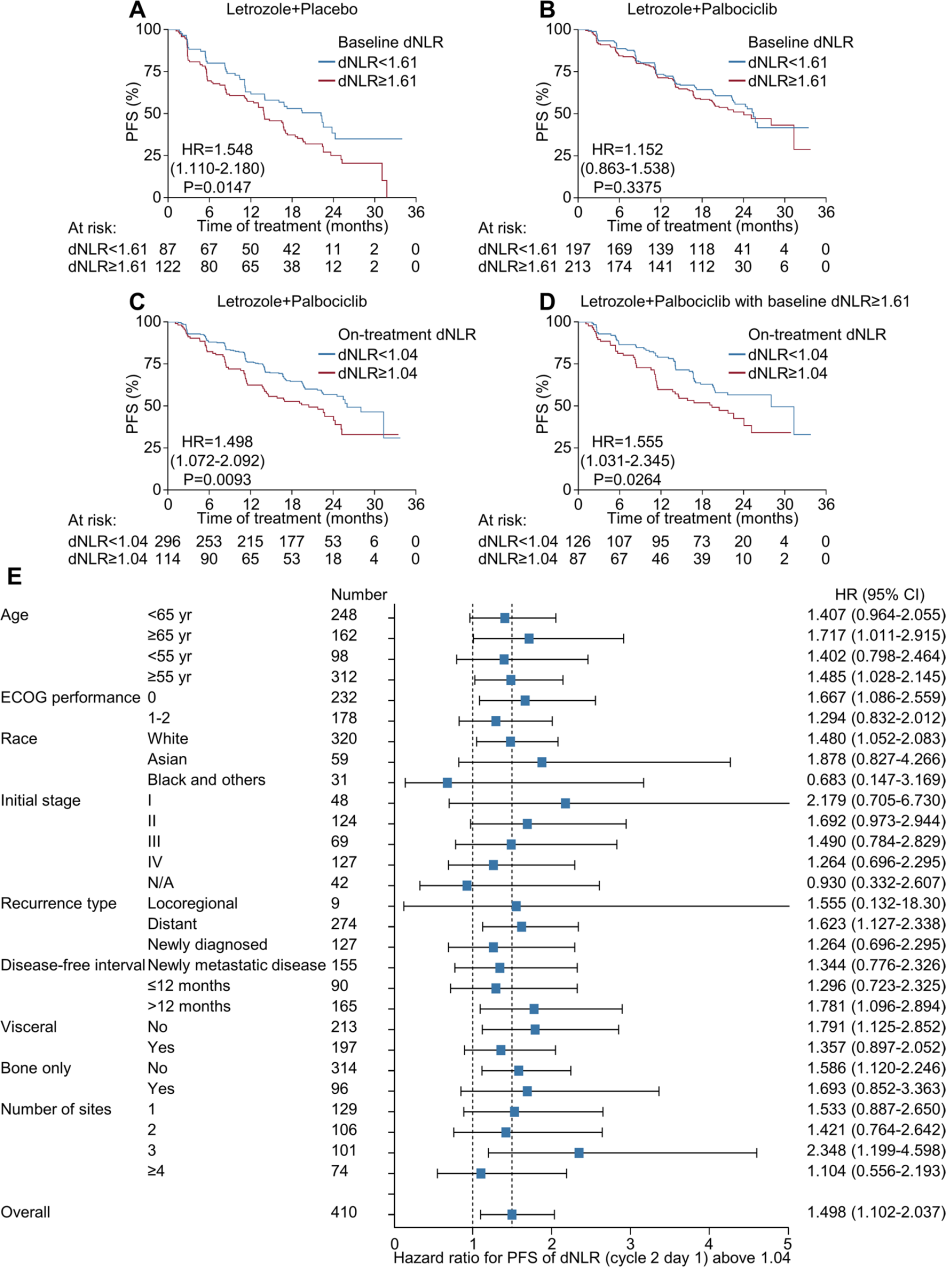
Survival outcomes based on changes in peripheral immune cell populations in the PALOMA-2 validation cohort. **A**, **B** Progression-free survival according to the dNLR ratio (cut-off = 1.61) at baseline in letrozole plus placebo-treated (*N* = 209) (**A**) or letrozole plus palbociclib-treated (*N* = 410) (**B**) patients. **C**, **D** Progression-free survival according to the dNLR ratio (cut-off = 1.04) after treatment (cycle 2 day 1) in letrozole plus palbociclib-treated patients (**C**) and in letrozole plus palbociclib-treated patients with higher baseline dNLR ratios (cut-off = 1.61) (**D**). **E** Forest plot of the dNLR ratio on progression-free survival in the validation cohort treated with letrozole plus palbociclib. CI, confidence interval; dNLR, derived neutrophil-to-lymphocyte ratio; ECOG, Eastern Cooperative Oncology Group; HR, hazard ratio; PFS, progression-free survival

Next, we assessed the predictive value of baseline and on-treatment dNLRs in both letrozole plus placebo- and letrozole plus palbociclib-treated patients. In patients receiving letrozole plus placebo, a higher dNLR at baseline (≥ 1.61) was associated with worse survival outcomes (Fig. 3A). In contrast, baseline dNLRs were not associated with PFS in patients receiving letrozole plus palbociclib (Fig. 3B); however, on-treatment dNLRs above 1.04 after letrozole plus palbociclib treatment were significantly associated with worse survival outcomes (HR for PFS = 1.498, *P* = 0.009, Fig. 3C) as well as a reduced likelihood of experiencing clinical benefit (Additional file 1: Table S11), although the HR for PFS was lower than that observed in the exploratory cohort. We further analyzed patients according to both baseline dNLR (cut-off 1.61) and on-treatment dNLR (cut-off 1.04). Letrozole plus palbociclib treatment significantly increased the frequency of the patients with lower on-treatment dNLRs (< 1.04) compared to letrozole plus placebo treatment (Additional file 1: Table S12), prompting us to investigate whether the lowering of dNLRs upon palbociclib treatment has predictive value. In patients with higher baseline dNLRs (≥ 1.61), a reduction of the on-treatment dNLR (< 1.04) was associated with favorable PFS (Fig. 3D), revealing that conversion of dNLR status can predict the survival benefit of palbociclib. Nevertheless, all dNLR-defined subgroups exhibited better PFS than patients subjected to letrozole monotherapy, and there was no interaction between the dNLR and treatment arm, suggesting that dNLR status is not a determinant for selecting between endocrine treatment or endocrine treatment plus CDK4/6 inhibitor therapy. The subgroup analysis demonstrated that the predictive value of on-treatment dNLRs was consistent irrespective of baseline characteristics (Fig. 3E). Multivariate analysis also revealed that on-treatment dNLRs independently predicted the survival outcomes of patients treated with letrozole plus palbociclib (Table 3); this was not observed in patients treated with letrozole plus placebo (Additional file 1: Table S13). Moreover, age, ECOG performance, and disease characteristics were prognostic factors of ABC rather than predictive factors of survival with palbociclib treatment, as can be deduced from the analysis of patients who received the placebo.

**Table 3:** Univariate and multivariate analyses of progression-free survival in validation cohort treated with letrozole and palbociclib

Index	Univariate analysis		Multivariate analysis	
	HR (95% CI)	*P* value	HR (95% CI)	*P* value
Age		0.040		0.029
≥ 65 years old	0.725 (0.533–0.985)		0.710 (0.522–0.966)	
< 65 years old	Reference		Reference	
Race		0.700		
White	Reference			
Others	0.931 (0.649–1.337)			
ECOG performance status		0.096		
0	Reference			
1, 2	1.280 (0.958–1.710)			
Initial stage		0.272		
III–IV	1.176 (0.881–1.570)			
Others	Reference			
Recurrence type		0.762		
Newly diagnosed	0.952 (0.694–1.306)			
Others	Reference			
Disease-free interval		0.398		
Newly metastatic disease	0.879 (0.653–1.185)			
Others	Reference			
Disease site		< 0.001		0.004
Visceral	1.838 (1.369–2.468)		1.661 (1.172–2.355)	
Nonvisceral	Reference		Reference	
Bone-only metastasis		0.008		0.239
Bone-only metastasis	0.601 (0.412–0.877)		0.763 (0.486–1.197)	
Others	Reference		Reference	
Number of disease sites		0.112		
≥ 3	1.265 (0.947–1.690)			
1, 2	Reference			
Derived neutrophil-to-lymphocyte ratio^a^		0.010		0.005
≥ 1.04	1.498 (1.102–2.037)		1.563 (1.148–2.129)	
< 1.04	Reference		Reference	

CI, confidence interval; ECOG, Eastern Cooperative Oncology Group; HR, hazard ratio

Next, we performed multiple log-rank tests with various on-treatment dNLR cut-off values to distinguish patient subgroups that would not benefit from treatment with letrozole plus palbociclib compared to letrozole alone (Additional file 8: Figure S7A). These analyses revealed that dNLR at cycle 2 day 1were inversely correlated with the benefit of palbociclib (Pearson *R*^2^ = 0.359, *P* < 0.001). When survival outcomes were measured in palbociclib-treated patients with extremely high on-treatment dNLRs (≥ 2.00; *N* = 17), no significant differences in PFS between patients treated with letrozole plus palbociclib and letrozole monotherapy were observed (Additional file 8: Figure S7B).

### Predictive value of dNLR in the PALOMA-3 study

To further validate the above findings, we analyzed the data from patients with HR-positive, HER2-negative ABC enrolled in the PALOMA-3 study who received fulvestrant treatment with or without palbociclib (Additional file 1: Table S14) [12]. Since the previous lines of treatment would affect the dNLR values in the PALOMA-3 study, we divided patients treated with fulvestrant plus palbociclib from the PALOMA-3 study into exploratory (*N* = 166) and validation (*N* = 166) cohorts. In the exploratory cohort, the cut-off value of on-treatment dNLR (0.88) for PFS prediction was determined using the log-rank maximization method. In both the exploratory and validation cohorts, the on-treatment dNLR was significantly associated with PFS (Additional file 9: Figure S8A-S8B). In subgroup analysis, the predictive value of the on-treatment dNLR was consistent irrespective of baseline characteristics (Additional file 9: Figure S8C). Furthermore, the on-treatment dNLR was independently associated with PFS in patients treated with fulvestrant and palbociclib (Additional file 1: Table S15), similarly to the results obtained using data from the PALOMA-2 study. Collectively, these results confirm that the on-treatment dNLR is a reliable marker for predicting palbociclib treatment outcomes.

### Analysis of circulating immune cells during treatment with CDK4/6 inhibitors

To evaluate the systemic effects of CDK4/6 inhibitors on immune cells, we serially obtained peripheral blood from patients with HR-positive, HER2-negative ABC who were administered CDK4/6 inhibitors (*N* = 20; Additional file 1: Table S16). First, we examined whether CDK4/6 inhibitors prevented T cell exhaustion by analyzing circulating CD8^+^ T lymphocytes. Intriguingly, the frequency of PD-1^+^ cells among CD8^+^ T lymphocytes was significantly attenuated after treatment with CDK4/6 inhibitors (Additional file 10: Figure S9A-S9B), suggesting that CDK4/6 inhibitors can systemically modulate T cell function [21, 22]. TOX is a crucial transcription factor for promoting T cell exhaustion [39], and NFATc1 is a main upstream regulator of TOX [32]; thus, we investigated the expression of these two transcription factors using flow cytometry. The expression levels of both TOX (Additional file 10: Figure S9C and S9F) and NFATc1 (Additional file 10: Figure S9D and S9F) in PD-1^+^CD8^+^ T lymphocytes were significantly decreased after treatment with CDK4/6 inhibitors, suggesting that CDK4/6 inhibitors modulate CD8^+^ T cell exhaustion both quantitatively and qualitatively. In line with this, the expression of CTLA-4 in PD-1^+^CD8^+^ T lymphocytes was also reduced after CDK4/6 administration (Additional file 10: Figure S9E-S9F). Because the increase in neutrophil counts in patients with cancer largely reflects a corresponding increase in MDSCs [27], we also checked the abundance of MDSCs relative to that of T cells. The ratio of MDSCs (including both M-MDSCs and PMN-MDSCs) to T cells was significantly decreased 15 days after administration of CDK4/6 inhibitors and slightly increased at cycle 2 day 1 compared to cycle 1 day 15 (Additional file 10: Figure S9G-S9H). However, the NLR was further reduced at cycle 2 day 1 compared to cycle 1 day 15 (Additional file 10: Figure S9I), indicating that changes in immune cell composition detected using flow cytometry predated those analyzed using routine laboratory tests. Consistent with this, the MDSC/T cell ratio on day 15 of cycle 1 was better correlated with the NLR value of cycle 2 day 1 compared to that of cycle 1 day 15 (Additional file 10: Figure S9J). Collectively, these results indicate that CDK4/6 inhibitors augment systemic antitumor immunity by preventing T cell exhaustion and diminishing the relative frequency of MDSCs, underscoring the translational relevance of immune monitoring during CDK4/6 inhibitor treatment.

## Discussion

CDK4/6 inhibitors have been established as a standard treatment for HR-positive, HER2-negative ABC, demonstrating clinical efficacy and safety in a series of clinical trials. Previous studies proposed that CDK4/6 inhibitors elicit robust antitumor immunity and that the response to CDK4/6 inhibitors essentially relies on their immunologic effects [40]; however, the role of dynamic immune biomarkers in predicting CDK4/6 inhibitor benefits had not been addressed. In this study, we demonstrated that the on-treatment (d)NLR was significantly reduced by CDK4/6 inhibitors and specifically predicted the benefits of letrozole plus palbociclib treatment. Mechanistically, we showed that pharmacologic inhibition of CDK4/6 promoted antitumor immunity by attenuating T cell exhaustion and MDSC abundance. This study sheds new light on the value of immune monitoring during treatment with CDK 4/6 inhibitors in patients with HR-positive, HER2-negative ABC.

The benefit of CDK4/6 inhibitors is consistent across patients with various clinical characteristics [9, 11, 20, 41]. In the PALOMA-3 study, cyclin E1 expression was associated with resistance to palbociclib combined with fulvestrant and with diminished antiproliferative effects after palbociclib treatment [13]. In the PALOMA-2 study, expression of receptor tyrosine kinases (e.g., FGFR2 or HER3) predicted palbociclib benefit [14]. In addition, various alterations (e.g., loss of *RB1*, estrogen receptor expression, and activating mutations in *AKT1*, *RAS*, *AURKA*, *CCNE2*, *ERBB2*, and *FGFR2*) mediate both intrinsic and acquired resistance to palbociclib [42]. Mutations in *RB1*, *PIK3CA*, and *ESR1* resulted in acquired resistance to palbociclib in the PALOMA-3 study [43]; however, these biomarkers were heterogeneous, explaining only a small portion of resistance mechanisms without a clear delineation between prognostic and predictive implications. In addition, next-generation sequencing was employed to explore the value of these biomarkers, which largely limited their usefulness and expandability in routine clinical practice. Therefore, apart from hormone receptor positivity, there are no clinically available biomarkers to identify patients who would benefit from CDK4/6 inhibitor treatment despite a clear clinical need.

In this study, we showed that palbociclib induced changes in immune cell composition, leading to a reduction in the dNLR after treatment. In addition, on-treatment neutrophil and lymphocyte counts were associated with palbociclib benefit in the opposite direction, necessarily giving predictive significance to the on-treatment (d)NLR in the exploratory cohort. The versatility of dNLRs to predict clinical outcomes was validated using data from letrozole plus palbociclib-treated patients enrolled in the PALOMA-2 study. Importantly, the PFS of patients treated with letrozole plus placebo was independent of the on-treatment dNLR value, indicating that the on-treatment dNLR is a predictive factor for palbociclib outcome rather than a simple prognostic factor. We also obtained concordant results in patients treated with fulvestrant and palbociclib in the PALOMA-3 study, although the on-treatment dNLR cut-off value was different due to marginally different on-treatment dNLR value between the patients enrolled in each study (Additional file 11: Figure S10A), probably can be attributed to patients’ disease status and exposure to previous therapies [44]. Additionally, our finding that CDK4/6 inhibitors modified the characteristics and numbers of lymphocytes and myeloid cells further supports the notion that response to CDK4/6 inhibitors depends on immune-modulating effects. Taken together, our on-treatment biomarkers showed strengths in general with independent external validation, were convenient and less invasive than current methods, and exhibited biological validity that reflects systemic immune responses. In line with our results, Zattarin et al. also reported that on-treatment NLRs and platelet-to-lymphocyte ratios after three cycles of treatment were associated with PFS [45].

In triple-negative and HER2-positive breast cancer, a high degree of tumor-infiltrating lymphocytes (TILs) is correlated with a low risk of relapse and with the effectiveness of chemotherapy [46–48]. However, increased TIL density is associated with shorter OS in HR-positive breast cancer patients [49], indicating a different underlying mechanism. Although HR-positive breast cancer is considered less immunogenic [50, 51], CDK4/6 inhibitors magnify immune recognition and the reaction of host immune cells toward tumor cells [52]. Of note, CDK4/6 inhibitors regulate PD-L1 stability in tumor cells [53], augment cytotoxic T cell activation [21], and attenuate suppressive functions of regulatory T cells [22], all of which contribute to robust antitumor immunity with reinforced host immune surveillance. These studies suggest that the mechanism of action of CDK4/6 inhibitors may involve mediation by host immune responses toward tumors. Our findings that CDK4/6 inhibitors prevented T cell exhaustion phenotypes via reducing PD-1, NFATc1, TOX, and CTLA-4 expression levels and MDSC numbers demonstrate that high dNLRs reflect active immune suppression with progressive T cell exhaustion in the tumor microenvironment, which is largely alleviated by CDK4/6 inhibitor treatment. A prospective study with larger cohorts of patients would help to evaluate further whether the on-treatment dNLR is a useful biomarker for predicting patient response to CDK4/6 inhibitor treatment. The utility of on-treatment dNLRs as a tool to measure systemic antitumor immune responses will be an interesting area of future study [54].

Our study provides a framework for future clinical trials and important clues for the interpreting several pivotal clinical trials. The PFS on palbociclib and letrozole therapy was superior to letrozole monotherapy regardless of on-treatment dNLR in our PALOMA-2 analysis, suggesting that (d)NLR status is not a determinant to select endocrine monotherapy or endocrine plus CDK4/6 inhibitor therapy. However, we think on-treatment dNLR can serve as important biomarker for drug selection and patient stratification for various clinical situations. In this regard, whether the (d)NLR can be used as a predictive marker for the benefit of adjuvant treatment with CDK4/6 inhibitors should be evaluated further. Recently, adjuvant trials with two different CDK4/6 inhibitors (NCT03155997 [monarchE] and NCT02513394 [PALLAS]) have shown conflicting results, highlighting the importance of identifying subgroups of patients that derive clinical benefits from CDK4/6 inhibitors. Our study revealed that patients with extremely high on-treatment dNLRs (≥ 2.00) did not derive benefit from adding palbociclib to letrozole treatment (Additional file 8: Figure S7A-S7B), although the number of these patients was small (17 of 410 patients treated with palbociclib and letrozole, 4.1%). Thus, our on-treatment biomarker may be essential tool for identifying these patient subgroups. Second, whether dose modification of CDK4/6 inhibitors has any effect on survival outcomes should be investigated. Dose reduction of CDK4/6 inhibitors is recommended in grade 3/4 hematologic adverse events, making it challenging to identify the dose–response relationship. Complicating infection accompanied by febrile neutropenia is rarely seen during treatment with CDK4/6 inhibitors, and continuing palbociclib treatment is safe in patients experiencing grade 3 neutropenia [55]. Thus, whether maintaining or increasing the dose of CDK4/6 inhibitors confers clinical benefits requires further investigation in future clinical trials because the NLR on cycle 2 day 1 may reflect the pharmacodynamic effects of palbociclib on bone marrow hematopoietic stem cells in addition to its immune-modulating effects. The third aspect of interest to future research is examining whether cytokines for T cell expansion (such as recombinant IL-7 or IL-15) restore lymphopenia and improve treatment responses to CDK4/6 inhibitors, because CDK4/6 inhibitor-related lymphopenia was associated with poor outcomes in our study. In addition, whether immune checkpoint inhibitor plus CDK4/6 inhibitor combinations elicit meaningful treatment responses should be investigated, especially in patients with HR-positive ABC, for whom several early phase studies are underway (NCT02778685, NCT02779751, and NCT02791334). We anticipate that these studies will show the clinical efficacy of these combined therapies, highlighting the immunomodulatory capacity of CDK4/6 inhibitors. Accordingly, we expect that on-treatment dNLR may guide optimal treatment sequence in HR-positive, HER2-negative ABC. Patients with high on-treatment (d)NLR is predicted to show poor response to CDK4/6 inhibitors, and they would be better candidates for early switching to cytotoxic chemotherapy and other novel therapies including targeted agents, antibody drug conjugates, or immunotherapy combined with CDK4/6 inhibitors. Lastly, the potential predictive value of on-treatment (d)NLR after CDK4/6 inhibitor therapy in terms of long-term OS needs to be explored. Considering that the results from long-term OS in three phase III studies (PALOMA-2, MONALEESA-2, and MONARCH-3) have been recently reported to be inconsistent, the influence of on-treatment (d)NLR on long-term OS outcome would give insights for more precise stratification of the patients.

The strengths of this study include the exploration and validation of clinically accessible biomarker for HR-positive, HER-2-negative ABC patients treated with CDK4/6 inhibitors, which was not observed in patients treated with only letrozole. In addition, immune correlates were explored along with translational relevance. Limitations to this study include the imbalances of baseline characteristics of the exploratory and validation cohort. Absence of lymphocyte counts in the validation cohort led us to use dNLR instead of NLR. Relatively small number of patients with immunologic analysis should be validated in a large scale with prospective manner. The statistical significance of on-treatment dNLR in the validation cohort was not as pronounced compared to that witnessed in the exploratory cohort.

## Conclusions

In summary, we discovered and validated that the on-treatment dNLR measured on day 1 of cycle 2 during palbociclib treatment significantly predicts survival outcomes of patients with HR-positive, HER2-negative ABC. Peripheral blood-based immune monitoring provides biological plausibility of systemic immune responses elicited by CDK4/6 inhibitors. Our results contribute to the identification of biomarkers for predicting CDK4/6 inhibitor treatment responses, the interpretation of completed and ongoing studies incorporating CDK4/6 inhibitors, and the design of upcoming clinical trials with translational relevance.

## Supplementary Information

**Additional file 1. Tables:** Table S1: List of reagents. Table S2: Univariate analysis of baseline and on treatment leukocyte ,neutrophil, and lymphocyte counts and their progression free survival. Table S3. Bivariate analysis of baseline and on treatment neutrophil tolymphocyte ratio s for progression free survival. Table S4. Bivariate analysis of baseline and on treatment derived neutrophilto lymphocyte ratio s for progression free survival. Table S5. C linical benefit probability according to derived neutrophil tolymphocyte ratio in the exploratory cohort. Table S6. Exploratory cohort baseline characteristics according to derivedneutrophil to lymphocyte ratio on day 1 of cycle 2. Supplementary Table S7 . Differences in derived neutrophil to lymphocyte ratio at cycle 2day 1 in the exploratory cohort with available next generation sequencing res ults according tospecific genetic alteration. Supplementary TableS 8 . Univariate and multivariate analyses of progression free survivalin the exploratory cohort with available next generation sequencing results according tospecific genetic alteration. Supplementary Table S9. Univariate and multivariate analyses for overall survival in the exploratory cohort. Supplementary Table S10. Baseline characteristics of the PALOMA 2 validation cohorttreated with letrozole plus palbociclib and derived neutrophil to lymphocyte ratio s at cycle 2day 1. Supplementary Table S11Probability of having clinical benefit according to the derivedneutrophil to lymphocyte ratio at cycle 2 day 1 in the PALOMA 2 validation cohort. Supplementary Table S12. Distribution of patients according to the derived neutrophil tolymphocyte ratio at baseline and cycle 2 day 1 in the PALOMA 2 validation cohort treated withletrozole with or without palbociclib. Supplementary Table S13. Univariate and multivariate analyses of progression free survivalof patients in the PALOMA 2 validation cohort treated with letrozole plus placebo. Supplementary Table S14. Baseline characteristics of patients in the PALOMA 3 cohorttreated with palbocic lib and fulvestrant with available derived neutrophil to lymphocyte ratio sat cycle 2 day 1. Supplementary Table S15. Univariate and multivariate analyses of progression free survival of patientsin the PALOMA 3 validation cohort treated with palbociclib and fulvestrant. Supplementary Table S16. Baseline characteristics of patients who underwent blood basedimmune monitoring.

**Additional file 2. FigureS1:** Dynamic changes in peripheral immune cell populationsafter palbociclib administration in the exploratory cohort ( N = (A C) Changes inleukocyte (A), neutrophil (B), and lymphocyte (C) counts before and after treatment (cycle 2day 1) (D F) Changes in leukocyte (D), neutrophil (E), and lymphocyte (F) counts aftertreatment (cycle 2 day 1) normalized to their respective baseline counts. (G) Fold change ofneutrophil and lymphocyte counts after treatment (cycle 2 day 1) compared to baseline (H I)Changes in the neutrophil to lymphocyte ratio before and after treatment (cycle 2 day 1) (H)and normalized to the baseline neutrophil to lymph ocyte ratio (

**Additional file 3. FigureS2:** Effect of treatment on neutrophil to lymphocyte ratios. (A B)Correlation between the neutrophil to lymphocyte ratio (A) and derived neutrophil tolymphocyte ratio (B) before and after treatment (cycle 2 day 1).

**Additional file 4. FigureS3:** Dynamic changes in derived neutrophil to lymphocyte ratio safter palbociclib administration in the exploratory cohort N =221). (A B) Changes in thederived neutrophil to lymphocyte ratio before and after treatment (cycle 2 day 1) (A) andnormalized to the baseline derived neutrophil to lymphocyte ratio (B). (C D) Correlationbetween the neutrophil to lymphocyte ratio and the derived neutrophil to lymphocyte ratio atbaseline (C) and after treatment (cycle 2 day 1) (D).

**Additional file 5. FigureS4:** Analysis of genetic alterationin the exploratory cohort withavailable next generation sequencing results (N=26). (A) Oncoplot for genetic

**Additional file 6. FigureS5:** Supplementary Figure S5. Dynamic changes in derived neutrophilto lymphocyte r atiosand survival outcomes according to on treatment derived neutrophil to lymphocyteratios after ribociclib administration (N=85). (A) Changes in the derived neutrophil tolymphocyte ratio before and after treatment (cycle 2 day 1). (B) Comparison of de rivedneutrophil to lymphocyte ratio after treatment (cycle 2 day 1) between patients administeredwith palbociclib (N=221) and ribociclib (N=85). (C) Progression free survival according tothe derived neutrophil to lymphocyte ratio (cut off=1.04) after tr eatment with ribociclib(cycle 2 day).

**Additional file 7. FigureS6:** C hanges in peripheral immune cell populations in thePALOMA 2 validation cohort. (A C) Changes in leukocyte (A) and neutrophil (B) countsand derived neutrophil to lymphocyte ratio (C) before and after treatment (cycle 2 day 1) inpatients treated with letrozole plus placebo (N= (D F) Changes in leukocyte (D) andneutrophil (E) counts and derived neutrophi l to lymphocyte ratio (F) before and aftertreatment (cycle 2 day 1) in patients treated with letrozole plus palbociclib (N= (G H)Progression free survival according to the derived neutrophil to lymphocyte ratio (cutoff=1.04) after treatment (cycle 2 day 1) in letrozole plus placebo treated patients. N.S., notsignificant.

**Additional file 8. FigureS7:** Outcome s of patients with extremely high on treatmentderived neutrophil to lymphocyte ratio s (cut off=2.00) (A) Plot of hazard ratio forprogression fre e survival of letrozole plus palbociclib treated patients above a specificderived neutrophil to lymphocyte ratio compared to that of letrozole plus placebo treatedpatients. (B) Progression free survival according to the derived neutrophil to lymphocyteratio (cut off=2.00) after treatment (cycle 2 day 1) in letrozole plus palbociclib treatedpatients compared to that of letrozole plus placebo treated patients. The h azard ratiocompar ed letrozole plus palbociclib treated p atients with an on treatment derived neutrophilto lymphocyte 2.00 and letrozole plus placebo treated patients. dNLR; derived neutrophilto lymphocyte ratio; HR, hazard ratio; PFS, progression free survival.

**Additional file 9. FigureS8:** Survival outcome s according to on treatment derivedneutrophil to lymphocyte ratio s in the PALOMA 3 cohort. (A B) Progression freesurvival according to the derived neutrophil to lymphocyte ratio (cut off=0.88) aftertreatment (cycle 2 day 1) in palbociclib and fulvestrant treated patients (N=332) from theexploratory cohort (A) and validation cohort (B). Patients were randomly assigned to theexploratory or validation cohort at a 1:1 ratio. (C) Forest plot of the derived neutrophil tolymphocyte ratio on progression free survival in the validation cohort treated with letrozoleplus palbociclib. CI, confidence interval; dNLR, derived neutrophil to lymphocyte ratio;ECOG, Eastern Cooperative Oncology Group; HR, hazar d ratio; PFS, progression freesurvival.

**Additional file 10. FigureS9:** C hanges in the characteristics and frequency of circulatingimmune cells after CDK4/6 inhibitor treatment ( N = (A B) Changes in the frequency of PD1 cells among CD8 T lymphocytes (A) and a representative histogram (B) (C F)Changes in the expression of NFATc1 (C), TOX (D), and CTLA 4 (E) in PD 1 CD8 Tlymphocytes and representative histograms (F) (G H) Changes in the relative frequency ofMDSC s and T cells (G) and visualization with t stochastic neighbor embedding (H) (I)Changes in the neutrophil to lymphocyte ratio (J) Correlation between the neutrophil tolymphocyte ratio and the MDSC to T cell ratio at the specified time points. C1D15, cycle 1day 15; C2D1, cycle 2 day 1; CTLA 4, cytotoxic T lymphocyte associated protein 4; MDSC,myeloid derived suppressor cell; MFI, mean fluorescent intensity; NFATc1, nuclear factor ofactivated T cells 1; PD 1, programmed death 1; TOX, thymocyte selection associated highmo bility group box protein.

**Additional file 11. FigureS10:** Supplementary Figure S10. Comparison of ontreatment derived neutrophil tolymphocyte ratios in the PALOMA 2 and 3 cohort administered with palbociclib. (A)Distribution of on treatment derived neutrophil to lymphocyte ratios in the PALOMA 2 and3 cohort administered with palbociclib.

## Data Availability

The datasets used and analyzed during the current study are available from the corresponding authors (exploratory cohort data) or Vivli, Inc (validation cohort data) upon reasonable request.
