# Supplementary material for: On-treatment derived neutrophil-to-lymphocyte ratio and survival with palbociclib and endocrine treatment: analysis of a multicenter retrospective cohort and the PALOMA-2/3 study with immune correlates
Source: Breast Cancer Res. 2023 Jan 12;25:4. doi: 10.1186/s13058-022-01601-4 (PMC9838072; doi:10.1186/s13058-022-01601-4)
Supplement: Supplementary file 1 — Additional file 1. Tables: Table S1: List of reagents. Table S2: Univariate analysis of baseline and on treatment leukocyte ,neutrophil, and lymphocyte counts and their progression free survival. Table S3. Bivariate analysis of baseline and on treatment neutrophil tolymphocyte ratio s for progression free survival. Table S4. Bivariate analysis of baseline and on treatment derived neutrophilto lymphocyte ratio s for progression free survival. Table S5. C linical benefit probability according to derived neutrophil tolymphocyte ratio in the exploratory cohort. Table S6. Exploratory cohort baseline characteristics according to derivedneutrophil to lymphocyte ratio on day 1 of cycle 2. Supplementary Table S7 . Differences in derived neutrophil to lymphocyte ratio at cycle 2day 1 in the exploratory cohort with available next generation sequencing res ults according tospecific genetic alteration. Supplementary TableS 8 . Univariate and multivariate analyses of progression free survivalin the exploratory cohort with available next generation sequencing results according tospecific genetic alteration. Supplementary Table S9. Univariate and multivariate analyses for overall survival in the exploratory cohort. Supplementary Table S10. Baseline characteristics of the PALOMA 2 validation cohorttreated with letrozole plus palbociclib and derived neutrophil to lymphocyte ratio s at cycle 2day 1. Supplementary Table S11Probability of having clinical benefit according to the derivedneutrophil to lymphocyte ratio at cycle 2 day 1 in the PALOMA 2 validation cohort. Supplementary Table S12. Distribution of patients according to the derived neutrophil tolymphocyte ratio at baseline and cycle 2 day 1 in the PALOMA 2 validation cohort treated withletrozole with or without palbociclib. Supplementary Table S13. Univariate and multivariate analyses of progression free survivalof patients in the PALOMA 2 validation cohort treated with letrozole plus placebo. Supplementary Table S14. Baseline [file 13058_2022_1601_MOESM1_ESM.docx]

**Supplementary Table S1.** List of reagents.

| **Reagent name** | **Manufacturer** | **Catalog number** |
| --- | --- | --- |
| Anti-CD-279 antibody | BioLegend | 329920 |
| Anti-CD14 antibody | BD Biosciences | 564054 |
| Anti-CD19 antibody | BD Biosciences | 562653 |
| Anti-CD8 antibody | BD Biosciences | 563677 |
| Anti-CD3 antibody | BD Biosciences | 563800 |
| Anti-CD4 antibody | BioLegend | 300530 |
| Anti-CD45RA antibody | BD Biosciences | 560673 |
| Anti-CD11b antibody | BioLegend | 301306 |
| Anti-NFATc1 antibody | BioLegend | 649606 |
| Anti-FoxP3 antibody | Thermo-Fisher Scientific | 12-4776-42 |
| Anti-CD14 antibody | BD Biosciences | 563743 |
| Anti-TOX antibody | Thermo-Fisher Scientific | 50-6502-82 |
| Anti-CD3 antibody | Thermo-Fisher Scientific | 25-0038-42 |
| Anti-Ki-67 antibody | BioLegend | 350522 |
| Anti-CD3 antibody | Thermo-Fisher Scientific | 25-0038-42 |
| Anti-CD152 antibody | BioLegend | 349914 |
| Anti-CD45 antibody | BioLegend | 368516 |
| Anti-CD33 antibody | BD Biosciences | 555626 |

**Supplementary Table S2.** Univariate analysis of baseline and on-treatment leukocyte, neutrophil, and lymphocyte counts and their progression-free survival.

| **Index** | **Hazard ratio (95% confidence interval)** | **P-value** |
| --- | --- | --- |
| Leucocyte (baseline) | 0.938 (0.837-1.052) | 0.277 |
| Neutrophil (baseline) | 0.981 (0.862-1.117) | 0.775 |
| Lymphocyte (baseline) | 0.689 (0.491-0.967) | 0.031 |
| Neutrophil-to-lymphocyte ratio (baseline) | 1.115 (0.992-1.253) | 0.069 |
| Derived neutrophil-to-lymphocyte ratio (baseline) | 1.210 (0.969-1.551) | 0.092 |
| Leucocyte (cycle 1 day 15) | 0.735 (0.539-1.003) | 0.052 |
| Neutrophil (cycle 1 day 15) | 1.082 (0.760-1.540) | 0.661 |
| Lymphocyte (cycle 1 day 15) | 0.626 (0.386-1.015) | 0.058 |
| Neutrophil-to-lymphocyte ratio (cycle 1 day 15) | 1.129 (0.927-1.375) | 0.227 |
| Derived neutrophil-to-lymphocyte ratio (cycle 1 day 15) | 0.961 (0.680-1.358) | 0.822 |
| Leucocyte (cycle 2 day 1) | 1.119 (0.940-1.333) | 0.207 |
| Neutrophil (cycle 2 day 1) | 1.453 (1.224-1.723) | <0.001 |
| Lymphocyte (cycle 2 day 1) | 0.636 (0.444-0.910) | 0.013 |
| Neutrophil-to-lymphocyte ratio (cycle 2 day 1) | 1.641 (1.399-1.926) | <0.001 |
| Derived neutrophil-to-lymphocyte ratio (cycle 2 day 1) | 2.524 (1.815-3.510) | <0.001 |

**Supplementary Table S3.** Bivariate analysis of baseline and on-treatment neutrophil-to-lymphocyte ratios for progression-free survival.

| **Index** | **Hazard ratio (95% confidence interval)** | **P-value** |
| --- | --- | --- |
| Neutrophil-to-lymphocyte ratio (baseline) | 1.070 (0.946-1.210) | 0.284 |
| Neutrophil-to-lymphocyte ratio (cycle 2 day 1) | 1.625 (1.380-1.915) | <0.001 |

**Supplementary Table S4.** Bivariate analysis of baseline and on-treatment derived neutrophil-to-lymphocyte ratios for progression-free survival.

| **Index** | **Hazard ratio (95% confidence interval)** | **P-value** |
| --- | --- | --- |
| Derived neutrophil-to-lymphocyte ratio (baseline) | 1.126 (0.890-1.425) | 0.321 |
| Derived neutrophil-to-lymphocyte ratio (cycle 2 day 1) | 2.473 (1.766-3.463) | <0.001 |

**Supplementary Table S5.** Clinical benefit probability according to derived neutrophil-to-lymphocyte ratio in the exploratory cohort.

| **Characteristics** | | **Total** | **On-treatment** | **On-treatment** | ***P* value** |
| --- | --- | --- | --- | --- | --- |
|  |  |  | **dNLR<1.04** | **dNLR≥1.04** |  |
|  |  | **(*N*=221)** | **(*N*=185)** | **(*N*=36)** |  |
| Clinical benefit | |  |  |  | 0.002 |
|  | Yes | 190 (86.0%) | 165 (89.2%) | 25 (69.4%) |  |
|  | No | 31 (14.0%) | 20 (10.8%) | 11 (30.6%) |  |
|  | | | | | |
| Sensitivity: 86.8% (165/190)  S | | | | | |
| Specificity: 35.5% (11/31) | | | | | |
| Positive predictive value: 89.2% (165/185) | | | | | |
| Negative predictive value: 30.6% (11/36) | | | | | |

Abbreviations: dNLR, derived neutrophil-to-lymphocyte ratio.

**Supplementary Table S6.** Exploratory cohort baseline characteristics according to derived neutrophil-to-lymphocyte ratio on day 1 of cycle 2.

| **Characteristics** | | **Total** | **On-treatment** | **On-treatment** | ***P* value** |
| --- | --- | --- | --- | --- | --- |
|  |  |  | **dNLR<1.04** | **dNLR≥1.04** |  |
|  |  | **(*N*=221)** | **(*N*=185)** | **(*N*=36)** |  |
| Age | |  |  |  | 0.550 |
|  | Median (range) | 55 (27-83) | 55 (27-83) | 58 (32-87) |  |
|  | <65 year old | 174 (78.7%) | 147 (79.5%) | 27 (75.0%) |  |
|  | ≥65 year old | 47 (21.3%) | 38 (20.5%) | 9 (25.0%) |  |
|  | <55 year old | 103 (46.6%) | 92 (49.7%) | 11 (30.6%) |  |
|  | ≥55 year old | 118 (53.4%) | 93 (50.3%) | 25 (69.4%) |  |
| Race | |  |  |  | 0.811 |
|  | White | 4 (1.8%) | 3 (1.6%) | 1 (2.8%) |  |
|  | Asian | 216 (97.7%) | 181 (97.8%) | 35 (97.2%) |  |
|  | Black | 0 (0.0%) | 0 (0.0%) | 0 (0.0%) |  |
|  | Other | 1 (0.5%) | 1 (0.5%) | 0 (0%) |  |
| Initial stage | |  |  |  | 0.382 |
|  | I | 31 (14.0%) | 25 (13.5%) | 6 (16.7%) |  |
|  | II | 66 (29.9%) | 56 (30.3%) | 10 (27.8%) |  |
|  | III | 26 (11.8%) | 22 (11.9%) | 4 (11.1%) |  |
|  | IV | 91 (41.2%) | 78 (42.2%) | 13 (36.1%) |  |
|  | N/A | 7 (3.2%) | 4 (2.2%) | 3 (8.3%) |  |
| Recurrence type | |  |  |  | 0.788 |
|  | Locoregional | 5 (2.3%) | 4 (2.2%) | 1 (2.8%) |  |
|  | Distant | 125 (56.6%) | 103 (55.7%) | 22 (61.1%) |  |
|  | Newly diagnosed | 91 (41.2%) | 78 (42.2%) | 13 (36.1%) |  |
| Disease-free interval | |  |  |  | 0.778 |
|  | Newly metastatic disease | 102 (46.2%) | 87 (47.0%) | 15 (41.7%) |  |
|  | ≤12 months | 44 (19.9%) | 37 (20.0%) | 7 (19.4%) |  |
|  | >12 months | 75 (33.9%) | 61 (33.0%) | 14 (38.9%) |  |
| Disease site | |  |  |  | 0.172 |
|  | Visceral | 123 (55.7%) | 78 (42.2%) | 20 (55.6%) |  |
|  | Nonvisceral | 98 (44.3%) | 107 (57.8%) | 16 (44.4%) |  |
|  | Bone-only | 44 (19.9%) | 33 (17.8%) | 11 (30.6%) |  |
| Number of disease sites | |  |  |  | 0.933 |
|  | 1 | 70 (31.7%) | 59 (31.9%) | 11 (30.6%) |  |
|  | 2 | 64 (29.0%) | 53 (28.6%) | 11 (30.6%) |  |
|  | 3 | 50 (22.6%) | 43 (23.2%) | 7 (19.4%) |  |
|  | ≥4 | 37 (16.7%) | 30 (16.2%) | 7 (19.4%) |  |

Abbreviations: dNLR, derived neutrophil-to-lymphocyte ratio; N/A, not assessed.

**Supplementary Table S7.** Differences in derived neutrophil-to-lymphocyte ratio at cycle 2 day 1 in the exploratory cohort with available next-generation sequencing results according to specific genetic alteration.

| **Genetic alteration** | **Presence** | **Absence** | **P-value**^a^ |
| --- | --- | --- | --- |
|  | Mean±SD^b^ | Mean±SD^b^ |  |
| *BRCA2* alteration | 0.657±0.355 | 0.602±0.433 | 0.835 |
| *CCND1* alteration | 0.473±0.128 | 0.627±0.441 | 0.561 |
| *CDH1* alteration | 0.436±0.156 | 0.623±0.432 | 0.554 |
| *CDKN1B* alteration | 0.579 | 0.610±0.427 | 0.944 |
| *CDKN2A/2B* alteration | 0.332 | 0.620±0.423 | 0.510 |
| *ERBB2* alteration | 0.766±0.426 | 0.588±0.423 | 0.500 |
| *ESR1* alteration | 0.380±0.113 | 0.639±0.435 | 0.323 |
| *FGF19* alteration | 0.430±0.148 | 0.624±0.432 | 0.541 |
| *FGF3* alteration | 0.430±0.148 | 0.624±0.432 | 0.541 |
| *FGF4* alteration | 0.430±0.148 | 0.624±0.432 | 0.541 |
| *FGFR1* alteration | 0.916±0.597 | 0.569±0.390 | 0.181 |
| *GATA3* alteration | 0.445±0.161 | 0.622±0.432 | 0.576 |
| *MDM2* alteration | 0.559 | 0.611±0.427 | 0.906 |
| *MSH3* alteration | 0.300 | 0.621±0.422 | 0.463 |
| *MTOR* alteration | 0.326 | 0.620±0.423 | 0.501 |
| *MYC* alteration | 0.657±0.515 | 0.594±0.399 | 0.754 |
| *PIK3CA* alteration | 0.600±0.467 | 0.620±0.373 | 0.905 |
| *PIK3R1* alteration | 0.579 | 0.610±0.427 | 0.944 |
| *PTEN* alteration | 0.368 | 0.618±0.424 | 0.568 |
| *ROHA* alteration | 0.326 | 0.620±0.423 | 0.501 |
| *RPS6KB* alteration | 0.433±0.144 | 0.623±0.432 | 0.548 |
| *SHQ1* alteration | 0.300 | 0.621±0.422 | 0.463 |
| *SMAD4* alteration | 0.476 | 0.614±0.426 | 0.752 |
| *SMO* alteration | 0.368 | 0.618±0.424 | 0.568 |
| *SPEN* alteration | 0.300 | 0.621±0.422 | 0.463 |
| *TP53* alteration | 0.736±0.582 | 0.516±0.220 | 0.192 |

Abbreviations: SD, standard deviation.

^a^Significance for difference

^b^Derived neutrophil-to-lymphocyte ratio at cycle 2 day 1

**Supplementary Table S8.** Univariate and multivariate analyses of progression-free survival in the exploratory cohort with available next-generation sequencing results according to specific genetic alteration.

| **Presence of alteration** | **Univariate analysis** | |  | **Multivariate analysis** | | |
| --- | --- | --- | --- | --- | --- | --- |
|  | **HR (95% CI)** | **P-value** |  | **HR (95% CI)** | **P-value** |  |
| *BRCA2* alteration (N=3) | 1.347 (0.290-6.266) | 0.704 |  |  |  |  |
| *CCND1* alteration (N=3) | 0.387 (0.050-2.980) | 0.362 |  |  |  |  |
| *CDH1* alteration (N=2) | 2.818 (0.606-13.100) | 0.186 |  |  |  |  |
| *CDKN1B* alteration (N=1) | 1.614 (0.204-12.763) | 0.650 |  |  |  |  |
| *CDKN2A/2B* alteration (N=1) | 24.495 (1.532-391.669) | 0.024 |  | 8.202 (0.491-136.936) | 0.143 |  |
| *ERBB2* alteration (N=3) | 0.347 (0.045-2.710) | 0.313 |  |  |  |  |
| *ESR1* alteration (N=3) | 1.573 (0.341-7.259) | 0.561 |  |  |  |  |
| *FGF19* alteration (N=2) | 0.472 (0.061-3.673) | 0.473 |  |  |  |  |
| *FGF3* alteration (N=2) | 0.472 (0.061-3.673) | 0.473 |  |  |  |  |
| *FGF4* alteration (N=2) | 0.472 (0.061-3.673) | 0.473 |  |  |  |  |
| *FGFR1* alteration (N=3) | 3.195 (0.640-15.947) | 0.157 |  |  |  |  |
| *GATA3* alteration (N=2) | 2.414 (0.295-19.773) | 0.411 |  |  |  |  |
| *MDM2* alteration (N=1) | 0.046 (0.000-105990.802) | 0.681 |  |  |  |  |
| *MSH3* alteration (N=1) | 0.045 (0.000-3182.889) | 0.586 |  |  |  |  |
| *MTOR* alteration (N=1) | 1.332 (0.169-10.483) | 0.786 |  |  |  |  |
| *MYC* alteration (N=6) | 6.842 (2.036-22.997) | 0.002 |  | 6.090 (1.726-21.489) | 0.005 |  |
| *PIK3CA* alteration (N=14) | 0.707 (0.245-2.037) | 0.520 |  |  |  |  |
| *PIK3R1* alteration (N=1) | 1.614 (0.204-12.763) | 0.650 |  |  |  |  |
| *PTEN* alteration (N=1) | 1.106 (0.141-8.670) | 0.924 |  |  |  |  |
| *ROHA* alteration (N=1) | 1.332 (0.169-10.483) | 0.786 |  |  |  |  |
| *RPS6KB* alteration (N=2) | 0.785 (0.099-6.189) | 0.818 |  |  |  |  |
| *SHQ1* alteration (N=1) | 0.045 (0.000-3182.889) | 0.586 |  |  |  |  |
| *SMAD4* alteration (N=1) | 2.364 (0.294-19.046) | 0.419 |  |  |  |  |
| *SMO* alteration (N=1) | 1.106 (0.141-8.670) | 0.924 |  |  |  |  |
| *SPEN* alteration (N=1) | 0.045 (0.000-3182.889) | 0.586 |  |  |  |  |
| *TP53* alteration (N=11) | 1.697 (0.584-4.929) | 0.331 |  |  |  |  |

Abbreviations: CI, confidence interval; HR, hazard ratio.

**Supplementary Table S9.** Univariate and multivariate analyses for overall survival in the exploratory cohort.

| **Index** | | **Univariate analysis** | |  | **Multivariate analysis** | |
| --- | --- | --- | --- | --- | --- | --- |
|  |  | **HR (95% CI)** | **P-value** |  | **HR (95% CI)** | **P-value** |
| Age | |  |  |  |  |  |
|  | ≥65 year old | 0.616 (0.238-1.598) | 0.319 |  |  |  |
|  | <65 year old | Reference |  |  |  |  |
| Race | |  |  |  |  |  |
|  | White | Reference |  |  |  |  |
|  | Others | 20.400 (0.000-Inf) | 0.766 |  |  |  |
| Initial stage | |  |  |  |  |  |
|  | III-IV | 1.288 (0.645-2.570) | 0.473 |  |  |  |
|  | Others | Reference |  |  |  |  |
| Recurrence type | |  |  |  |  |  |
|  | Newly diagnosed | 1.135 (0.569-2.265) | 0.719 |  |  |  |
|  | Others | Reference |  |  |  |  |
| Disease-free interval | |  |  |  |  |  |
|  | Newly metastatic disease | 1.055 (0.531-2.095) | 0.878 |  |  |  |
|  | Others | Reference |  |  |  |  |
| Disease site | |  |  |  |  |  |
|  | Visceral | 1.558 (0.754-3.216) | 0.231 |  |  |  |
|  | Nonvisceral | Reference |  |  |  |  |
| Bone-only metastasis | |  |  |  |  |  |
|  | Bone-only metastasis | 0.808 (0.333-1.959) | 0.638 |  |  |  |
|  | Others | Reference |  |  |  |  |
| Number of disease sites | |  |  |  |  |  |
|  | ≥3 | 2.904 (1.441-5.853) | 0.003 |  | 2.794 (1.388-5.623) | 0.004 |
|  | 1, 2 | Reference |  |  | Reference |  |
| Derived neutrophil-to-lymphocyte ratio^a^ | |  |  |  |  |  |
|  | ≥1.04 | 5.406 (2.719-10.749) | <0.001 |  | 5.242 (2.639-10.412) | <0.001 |
|  | <1.04 | Reference |  |  | Reference |  |

Abbreviations: CI, confidence interval; HR, hazard ratio.

^a^Measured at cycle 2 day 1

**Supplementary Table S10.** Baseline characteristics of the PALOMA-2 validation cohort treated with letrozole plus palbociclib and derived neutrophil-to-lymphocyte ratios at cycle 2 day 1.

| **Characteristics** | | **Total** | **On-treatment** | **On-treatment** | **P-value** |
| --- | --- | --- | --- | --- | --- |
|  |  |  | **dNLR<1.04** | **dNLR≥1.04** |  |
|  |  | **(N=410)** | **C2D1 (N=296)** | **(N=114)** |  |
| Age | |  |  |  | 0.847 |
|  | Median (range) | 62 (30-89) | 62 (30-86) | 61 (36-89) |  |
|  | <65 year old | 248 (60.5%) | 179 (60.5%) | 69 (60.5%) |  |
|  | ≥65 year old | 162 (39.5%) | 117 (39.5%) | 45 (39.5%) |  |
|  | <55 year old | 98 (23.9%) | 69 (23.3%) | 29 (25.4%) |  |
|  | ≥55 year old | 312 (76.1%) | 227 (76.7%) | 85 (74.6%) |  |
| ECOG performance status | |  |  |  | 0.135 |
|  | 0 | 232 (56.6%) | 173 (58.4%) | 59 (51.8%) |  |
|  | 1 | 171 (41.7%) | 120 (40.5%) | 51 (44.7%) |  |
|  | 2 | 7 (1.7%) | 3 (1.0%) | 4 (3.5%) |  |
| Race | |  |  |  | 0.361 |
|  | White | 320 (78%) | 227 (76.7%) | 93 (81.6%) |  |
|  | Asian | 59 (14.4%) | 44 (14.9%) | 15 (13.2%) |  |
|  | Black | 7 (1.7%) | 7 (2.4%) | 0 (0.0%) |  |
|  | Other | 24 (5.9%) | 18 (6.1%) | 6 (5.3%) |  |
| Initial stage | |  |  |  | 0.078 |
|  | I | 48 (11.7%) | 40 (13.5%) | 8 (7.0%) |  |
|  | II | 124 (30.2%) | 87 (29.4%) | 37 (32.5%) |  |
|  | III | 69 (16.8%) | 42 (14.2%) | 27 (23.7%) |  |
|  | IV | 127 (31.0%) | 96 (32.4%) | 31 (27.2%) |  |
|  | N/A | 42 (10.2%) | 31 (10.5%) | 11 (9.6%) |  |
| Recurrence type | |  |  |  | 0.586 |
|  | Locoregional | 9 (2.2%) | 6 (2.0%) | 3 (2.6%) |  |
|  | Distant | 274 (66.8%) | 194 (65.5%) | 80 (70.2%) |  |
|  | Newly diagnosed | 127 (31.0%) | 96 (32.4%) | 31 (27.2%) |  |
| Disease-free interval | |  |  |  |  |
|  | Newly metastatic disease | 155 (37.8%) | 116 (39.2%) | 39 (34.2%) |  |
|  | ≤12 months | 90 (22.0%) | 62 (20.9%) | 28 (24.6%) |  |
|  | >12 months | 165 (40.2%) | 118 (39.9%) | 47 (41.2%) |  |
| Disease site | |  |  |  | 0.552 |
|  | Visceral | 197 (48.0%) | 143 (48.3%) | 54 (47.4%) |  |
|  | Nonvisceral | 213 (52.0%) | 153 (51.7%) | 60 (52.6%) |  |
|  | Bone-only | 96 (23.4%) | 64 (21.6%) | 32 (28.1%) |  |
| Number of disease sites | |  |  |  | 0.424 |
|  | 1 | 129 (31.5%) | 87 (29.4%) | 42 (36.8%) |  |
|  | 2 | 106 (25.9%) | 76 (25.7%) | 30 (26.3%) |  |
|  | 3 | 101 (24.6%) | 76 (25.7%) | 25 (21.9%) |  |
|  | ≥4 | 74 (18.0%) | 57 (19.3%) | 17 (14.9%) |  |

Abbreviations: dNLR, derived neutrophil-to-lymphocyte ratio; ECOG, Eastern Cooperative Oncology Group; N/A, not assessed.

**Supplementary Table S11.** Probability of having clinical benefit according to the derived neutrophil-to-lymphocyte ratio at cycle 2 day 1 in the PALOMA-2 validation cohort.

| **Characteristics** | | **Total** | **On-treatment** | **On-treatment** | ***P* value** |
| --- | --- | --- | --- | --- | --- |
|  |  |  | **dNLR<1.04** | **dNLR≥1.04** |  |
|  |  | **(*N*=410)** | **(*N*=296)** | **(*N*=114)** |  |
| Clinical benefit | |  |  |  | 0.030 |
|  | Yes | 355 (86.6%) | 263 (88.9%) | 92 (80.7%) |  |
|  | No | 55 (13.4%) | 33 (11.1%) | 22 (19.3%) |  |
|  | | | | | |
| Sensitivity: 74.1% (263/355)  S | | | | | |
| Specificity: 40.0% (22/55) | | | | | |
| Positive predictive value: 88.9% (263/296) | | | | | |
| Negative predictive value: 19.3% (22/114) | | | | | |

Abbreviations: dNLR, derived neutrophil-to-lymphocyte ratio.

**Supplementary Table S12.** Distribution of patients according to the derived neutrophil-to-lymphocyte ratio at baseline and cycle 2 day 1 in the PALOMA-2 validation cohort treated with letrozole with or without palbociclib.

| **Characteristics** | | **Total** | **Letrozole plus** | **Letrozole plus** | **P-value** |
| --- | --- | --- | --- | --- | --- |
|  |  |  | **placebo** | **palbociclib** |  |
|  |  | **(N=619)** | **(N=209)** | **(N=410)** |  |
| Baseline dNLR | |  |  |  | 0.129 |
|  | <1.61 | 284 (45.9%) | 87 (41.6%) | 197 (48.0%) |  |
|  | ≥1.61 | 335 (54.1%) | 122 (58.4%) | 213 (52.0%) |  |
| On-treatment dNLR | |  |  |  | <0.001 |
|  | <1.04 | 328 (53.0%) | 32 (15.3%) | 296 (72.2%) |  |
|  | ≥1.04 | 291 (47.0%) | 177 (84.7%) | 114 (27.8%) |  |

Abbreviations: dNLR, derived neutrophil-to-lymphocyte ratio.

**Supplementary Table S13.** Univariate and multivariate analyses of progression-free survival of patients in the PALOMA-2 validation cohort treated with letrozole plus placebo.

| **Index** | | **Univariate analysis** | |  | **Multivariate analysis** | |
| --- | --- | --- | --- | --- | --- | --- |
|  |  | **HR (95% CI)** | **P-value** |  | **HR (95% CI)** | **P-value** |
| Age | |  | 0.090 |  |  |  |
|  | ≥65 year old | 0.723 (0.497-1.052) |  |  |  |  |
|  | <65 year old | Reference |  |  |  |  |
| Race | |  | 0.920 |  |  |  |
|  | White | Reference |  |  |  |  |
|  | Others | 1.021 (0.678-1.538) |  |  |  |  |
| ECOG | |  | 0.011 |  |  | 0.003 |
|  | 0 | Reference |  |  | Reference |  |
|  | 1, 2 | 1.581 (1.112-2.247) |  |  | 1.718 (1.202-2.455) |  |
| Initial stage | |  | 0.040 |  |  | 0.485 |
|  | III-IV | 0.697 (0.494-0.984) |  |  | 0.848 (0.534-1.347) |  |
|  | Others | Reference |  |  | Reference |  |
| Recurrence type | |  | 0.025 |  |  | 0.120 |
|  | Newly diagnosed | 0.641 (0.435-0.945) |  |  | 0.660 (0.391-1.114) |  |
|  | Others | Reference |  |  | Reference |  |
| Disease-free interval | |  | 0.968 |  |  |  |
|  | Newly metastatic disease | 0.993 (0.703-1.403) |  |  |  |  |
|  | Others | Reference |  |  |  |  |
| Disease site | |  | 0.054 |  |  |  |
|  | Visceral | 1.400 (0.994-1.972) |  |  |  |  |
|  | Nonvisceral | Reference |  |  |  |  |
| Bone-only metastasis | |  | 0.352 |  |  |  |
|  | Bone-only metastasis | 1.217 (0.805-1.840) |  |  |  |  |
|  | Others | Reference |  |  |  |  |
| Number of disease sites | |  | 0.283 |  |  |  |
|  | ≥3 | 1.206 (0.857-1.697) |  |  |  |  |
|  | 1, 2 | Reference |  |  |  |  |
| Derived neutrophil-to-lymphocyte ratio^a^ | |  | 0.179 |  |  |  |
|  | ≥1.04 | 1.419 (0.852-2.365) |  |  |  |  |
|  | <1.04 | Reference |  |  |  |  |

Abbreviations: CI, confidence interval; ECOG, Eastern Cooperative Oncology Group; HR, hazard ratio.

^a^Measured at cycle 2 day 1

**Supplementary Table S14.** Baseline characteristics of patients in the PALOMA-3 cohort treated with palbociclib and fulvestrant with available derived neutrophil-to-lymphocyte ratios at cycle 2 day 1.

| **Characteristics** | | **Total** |
| --- | --- | --- |
|  |  | **(N=332)** |
| Age | |  |
|  | Median (range) | 57 (30-88) |
|  | <65 year old | 249 (75.0%) |
|  | ≥65 year old | 83 (25.0%) |
|  | <55 year old | 142 (42.8%) |
|  | ≥55 year old | 190 (57.2%) |
| ECOG performance status | |  |
|  | 0 | 197 (59.3%) |
|  | 1 | 135 (40.7%) |
| Race | |  |
|  | White | 243 (73.2%) |
|  | Asian | 69 (20.8%) |
|  | Black | 11 (3.3%) |
|  | Other | 9 (2.7%) |
| Menopausal status | |  |
|  | Premenopausal or perimenopausal | 66 (19.9%) |
|  | Postmenopausal | 266 (80.1%) |
| Measurable disease | |  |
|  | Yes | 254 (76.5%) |
|  | No | 78 (23.5%) |
| Visceral disease | |  |
|  | Yes | 193 (58.1%) |
|  | No | 139 (41.9%) |
| Number of disease sites | |  |
|  | 1 | 111 (33.4%) |
|  | 2 | 95 (28.6%) |
|  | 3 | 69 (20.8%) |
|  | ≥4 | 57 (17.2%) |
| Disease-free interval | |  |
|  | Data available | 218 (65.7%) |
|  | >24 months | 182 (54.8%) |
|  | 12-24 months | 25 (7.5%) |
|  | <12 months | 11 (3.3%) |
| Number of previous line of treatment for advanced disease | |  |
|  | 0 | 82 (24.7%) |
|  | 1 | 126 (38.0%) |
|  | 2 | 85 (25.6%) |
|  | ≥3 | 39 (11.7%) |

**Supplementary Table S15.** Univariate and multivariate analyses of progression-free survival of patients in the PALOMA-3 validation cohort treated with palbociclib and fulvestrant.

| **Index** | | **Univariate analysis** | |  | **Multivariate analysis** | |
| --- | --- | --- | --- | --- | --- | --- |
|  |  | **HR (95% CI)** | **P-value** |  | **HR (95% CI)** | **P-value** |
| Age | |  | 0.333 |  |  |  |
|  | ≥65 year old | 0.786 (0.483-1.279) |  |  |  |  |
|  | <65 year old | Reference |  |  |  |  |
| ECOG | |  | 0.109 |  |  |  |
|  | 0 | Reference |  |  |  |  |
|  | 1 | 1.396 (0.928-2.100) |  |  |  |  |
| Race | |  | 0.732 |  |  |  |
|  | White | Reference |  |  |  |  |
|  | Others | 0.921 (0.573-1.478) |  |  |  |  |
| Menopausal status | |  | 0.816 |  |  |  |
|  | Premenopausal or perimenopausal | Reference |  |  |  |  |
|  | Postmenopausal | 1.065 (0.629-1.803) |  |  |  |  |
| Measurable disease | |  | 0.042 |  |  | 0.641 |
|  | Yes | 1.751 (1.021-3.004) |  |  | 1.184 (0.582-2.407) |  |
|  | No | Reference |  |  | Reference |  |
| Visceral disease | |  | 0.016 |  |  | 0.291 |
|  | Yes | 1.720 (1.109-2.667) |  |  | 1.371 (0.762-2.467) |  |
|  | No | Reference |  |  | Reference |  |
| Number of disease sites | |  | 0.007 |  |  | 0.108 |
|  | ≥3 | 1.763 (1.172-2.652) |  |  | 1.471 (0.918-2.356) |  |
|  | 1, 2 | Reference |  |  | Reference |  |
| Disease-free interval | |  | 0.008 |  |  | 0.016 |
|  | <24 months | 2.130 (1.220-3.719) |  |  | 2.018 (1.142-3.566) |  |
|  | >24 months or unknown | Reference |  |  | Reference |  |
| Line of treatment | |  | 0.085 |  |  |  |
|  | ≥3 | 1.440 (0.951-2.182) |  |  |  |  |
|  | 1, 2 | Reference |  |  |  |  |
| Derived neutrophil-to-lymphocyte ratio^a^ | |  | 0.004 |  |  | 0.005 |
|  | ≥0.88 | 1.824 (1.212-2.745) |  |  | 1.808 (1.191-2.747) |  |
|  | <0.88 | Reference |  |  | Reference |  |

Abbreviations: CI, confidence interval; ECOG, Eastern Cooperative Oncology Group; HR, hazard ratio.

^a^Measured at cycle 2 day 1

**Supplementary Table S16.** Baseline characteristics of patients who underwent blood-based immune monitoring.

| **Characteristics** | | **Total** |
| --- | --- | --- |
|  |  | **(N=20)** |
| Age | |  |
|  | Median | 53 (38-74) |
| Race | |  |
|  | Asian | 20 (100.0%) |
| Initial stage | |  |
|  | I | 4 (20.0%) |
|  | II | 8 (40.0%) |
|  | III | 3 (15.0%) |
|  | IV | 5 (25.0%) |
| Recurrence type | |  |
|  | Distant | 15 (75.0%) |
|  | Newly diagnosed | 5 (25.0%) |
| Disease-free interval | |  |
|  | Newly metastatic disease | 5 (25.0%) |
|  | ≤12 months | 5 (25.0%) |
|  | >12 months | 10 (50.0%) |
| Disease site | |  |
|  | Visceral | 13 (65.0%) |
|  | Nonvisceral | 5 (25.0%) |
|  | Bone-only | 2 (10.0%) |
| Number of disease sites | |  |
|  | 1 | 4 (20.0%) |
|  | 2 | 9 (45.0%) |
|  | 3 | 4 (20.0%) |
|  | ≥4 | 3 (15.0%) |
