# Supplementary figures and images for: On-treatment derived neutrophil-to-lymphocyte ratio and survival with palbociclib and endocrine treatment: analysis of a multicenter retrospective cohort and the PALOMA-2/3 study with immune correlates
Source: Breast Cancer Res. 2023 Jan 12;25:4. doi: 10.1186/s13058-022-01601-4 (PMC9838072; doi:10.1186/s13058-022-01601-4)

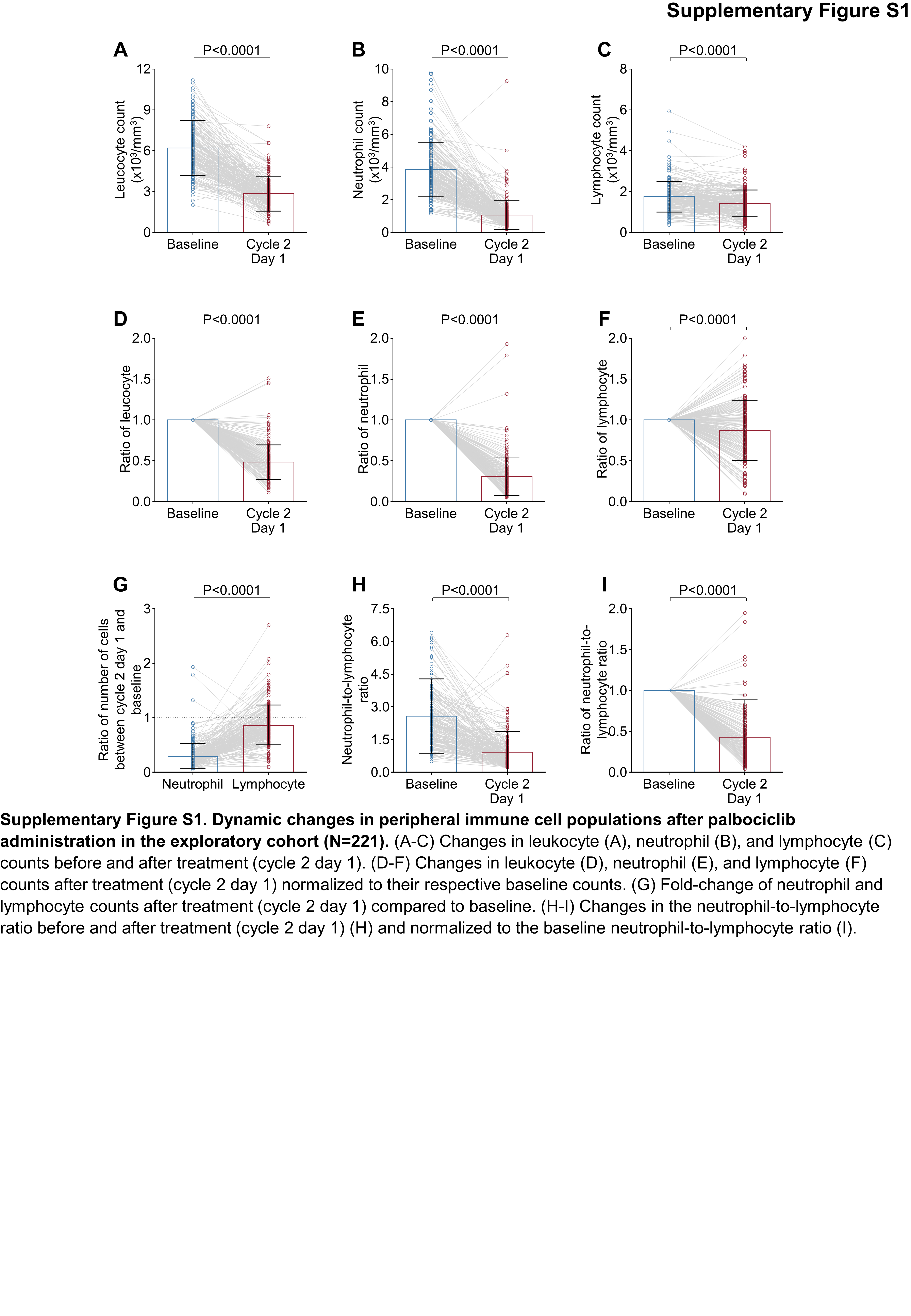

Supplement: Supplementary file 2 — Additional file 2. FigureS1: Dynamic changes in peripheral immune cell populationsafter palbociclib administration in the exploratory cohort ( N = (A C) Changes inleukocyte (A), neutrophil (B), and lymphocyte (C) counts before and after treatment (cycle 2day 1) (D F) Changes in leukocyte (D), neutrophil (E), and lymphocyte (F) counts aftertreatment (cycle 2 day 1) normalized to their respective baseline counts. (G) Fold change ofneutrophil and lymphocyte counts after treatment (cycle 2 day 1) compared to baseline (H I)Changes in the neutrophil to lymphocyte ratio before and after treatment (cycle 2 day 1) (H)and normalized to the baseline neutrophil to lymph ocyte ratio ( [file 13058_2022_1601_MOESM2_ESM.tif]

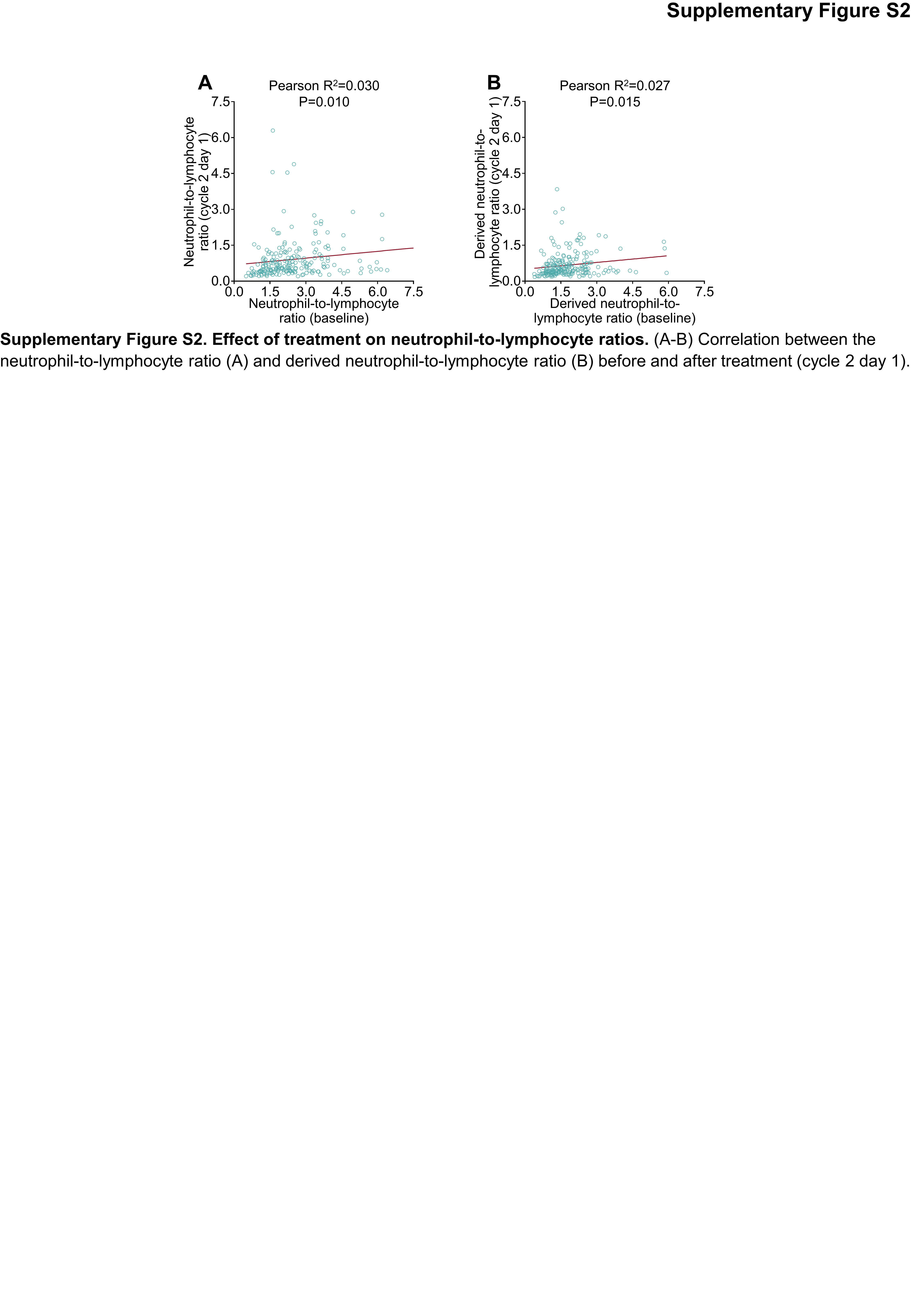

Supplement: Supplementary file 3 — Additional file 3. FigureS2: Effect of treatment on neutrophil to lymphocyte ratios. (A B)Correlation between the neutrophil to lymphocyte ratio (A) and derived neutrophil tolymphocyte ratio (B) before and after treatment (cycle 2 day 1). [file 13058_2022_1601_MOESM3_ESM.tif]

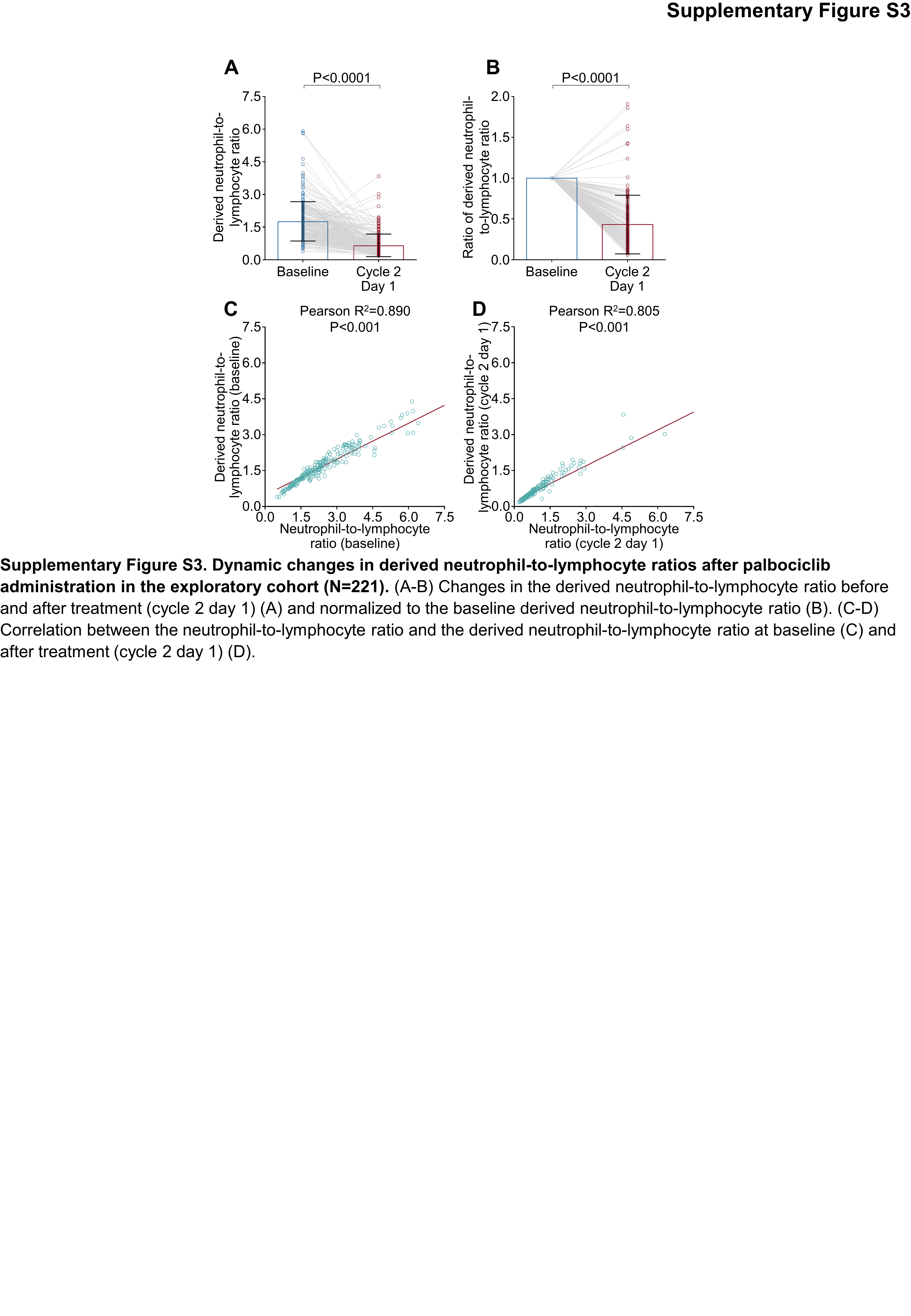

Supplement: Supplementary file 4 — Additional file 4. FigureS3: Dynamic changes in derived neutrophil to lymphocyte ratio safter palbociclib administration in the exploratory cohort N =221). (A B) Changes in thederived neutrophil to lymphocyte ratio before and after treatment (cycle 2 day 1) (A) andnormalized to the baseline derived neutrophil to lymphocyte ratio (B). (C D) Correlationbetween the neutrophil to lymphocyte ratio and the derived neutrophil to lymphocyte ratio atbaseline (C) and after treatment (cycle 2 day 1) (D). [file 13058_2022_1601_MOESM4_ESM.tif]

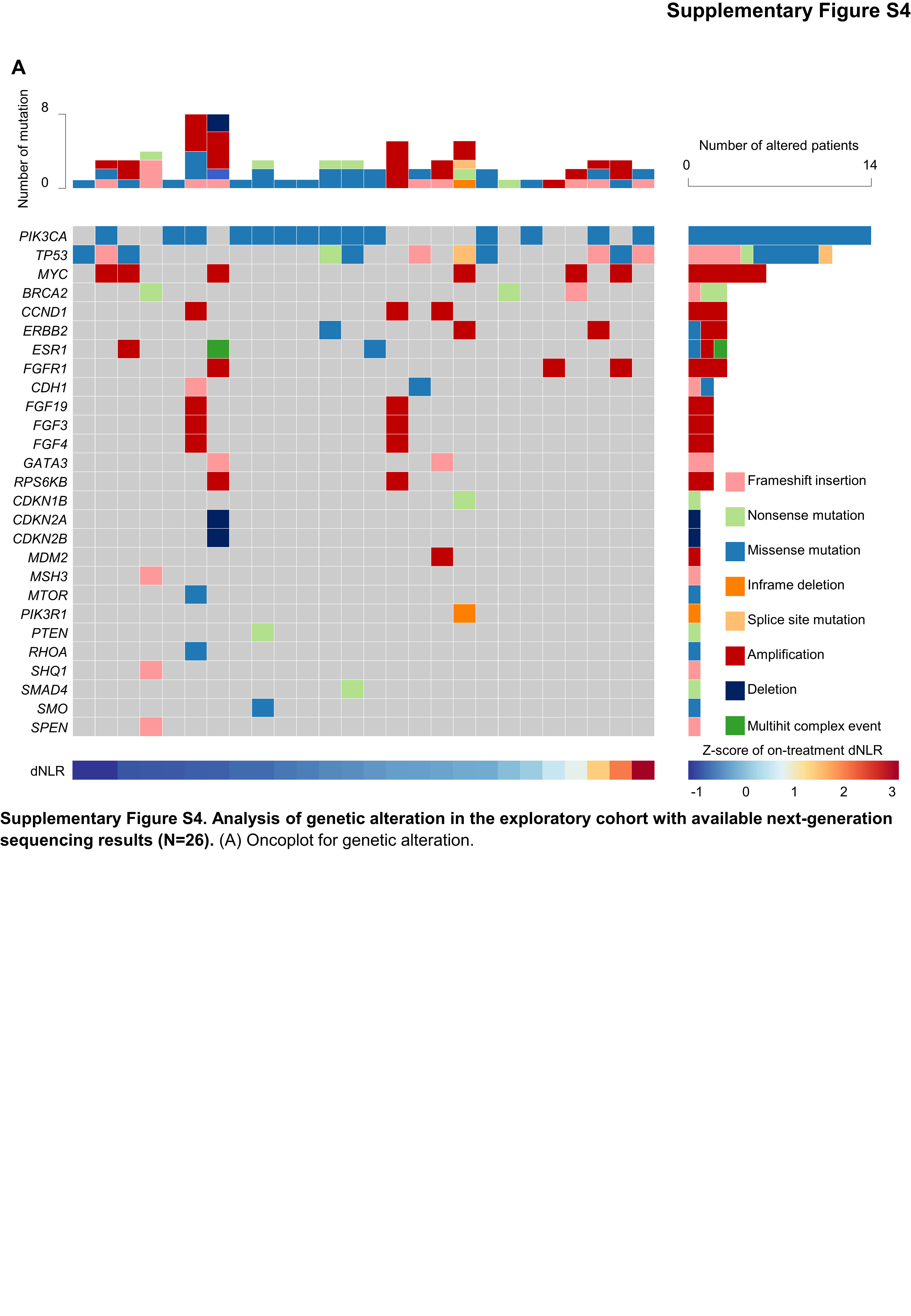

Supplement: Supplementary file 5 — Additional file 5. FigureS4: Analysis of genetic alterationin the exploratory cohort withavailable next generation sequencing results (N=26). (A) Oncoplot for genetic [file 13058_2022_1601_MOESM5_ESM.tif]

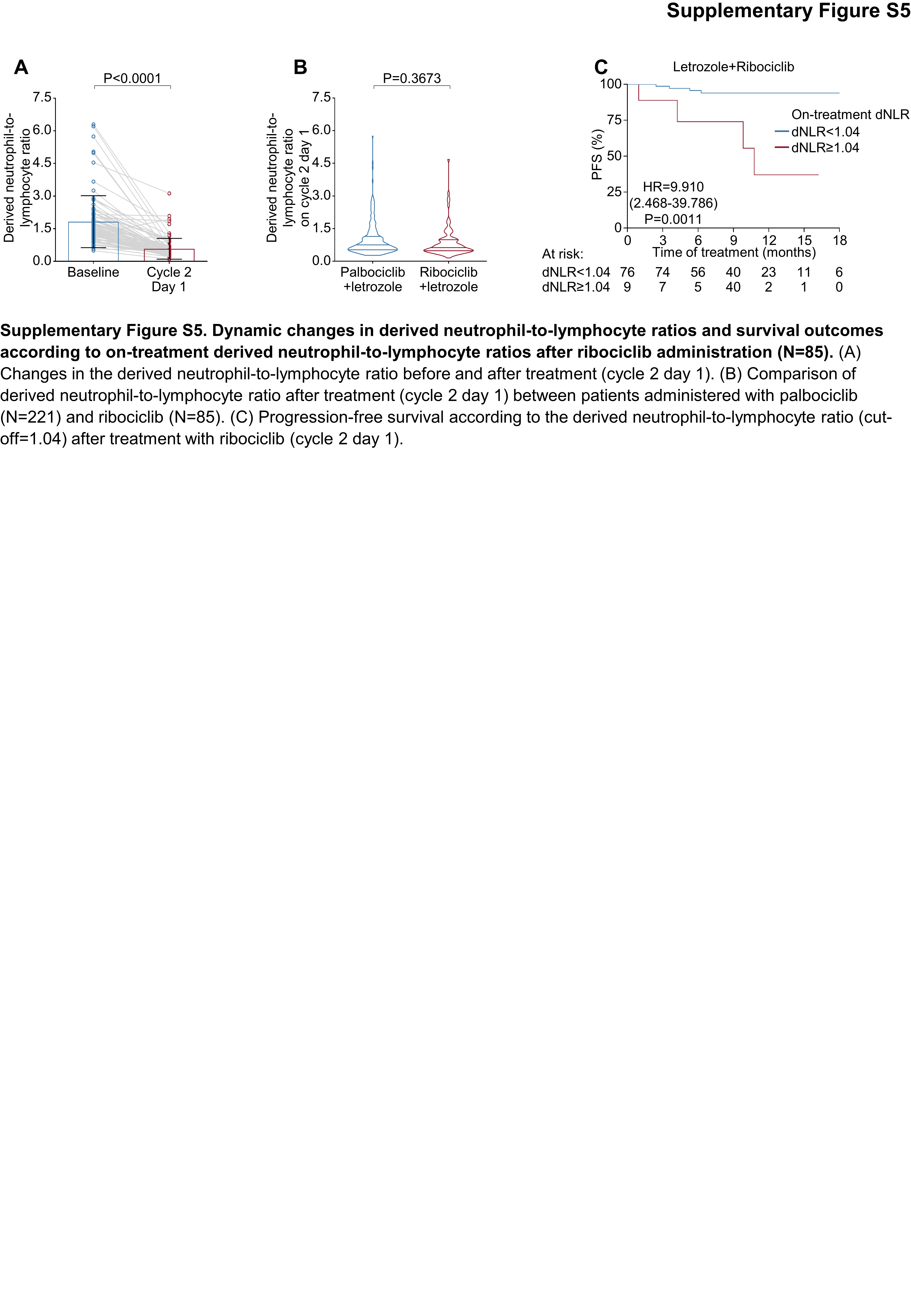

Supplement: Supplementary file 6 — Additional file 6. FigureS5: Supplementary Figure S5. Dynamic changes in derived neutrophilto lymphocyte r atiosand survival outcomes according to on treatment derived neutrophil to lymphocyteratios after ribociclib administration (N=85). (A) Changes in the derived neutrophil tolymphocyte ratio before and after treatment (cycle 2 day 1). (B) Comparison of de rivedneutrophil to lymphocyte ratio after treatment (cycle 2 day 1) between patients administeredwith palbociclib (N=221) and ribociclib (N=85). (C) Progression free survival according tothe derived neutrophil to lymphocyte ratio (cut off=1.04) after tr eatment with ribociclib(cycle 2 day). [file 13058_2022_1601_MOESM6_ESM.tif]

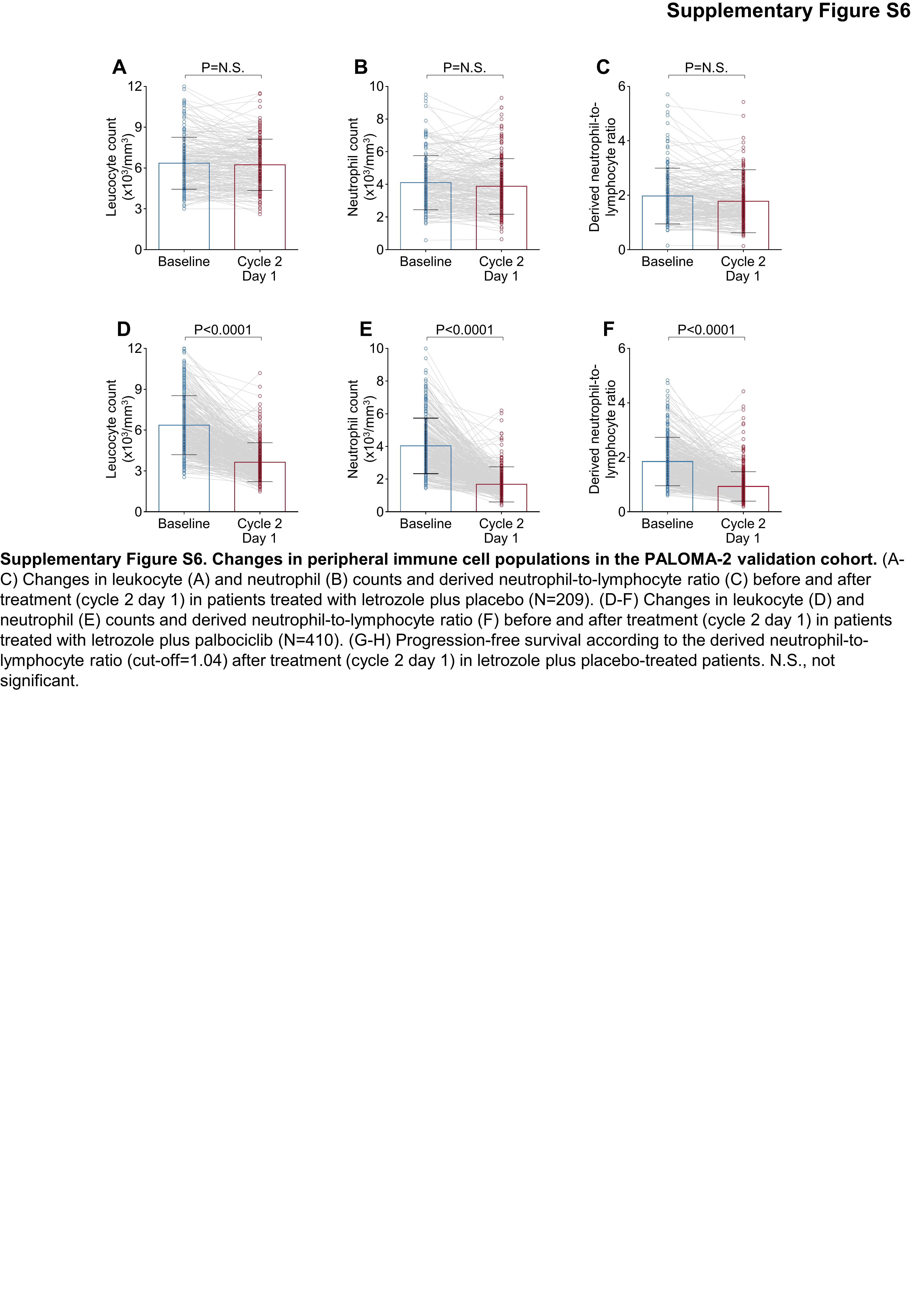

Supplement: Supplementary file 7 — Additional file 7. FigureS6: C hanges in peripheral immune cell populations in thePALOMA 2 validation cohort. (A C) Changes in leukocyte (A) and neutrophil (B) countsand derived neutrophil to lymphocyte ratio (C) before and after treatment (cycle 2 day 1) inpatients treated with letrozole plus placebo (N= (D F) Changes in leukocyte (D) andneutrophil (E) counts and derived neutrophi l to lymphocyte ratio (F) before and aftertreatment (cycle 2 day 1) in patients treated with letrozole plus palbociclib (N= (G H)Progression free survival according to the derived neutrophil to lymphocyte ratio (cutoff=1.04) after treatment (cycle 2 day 1) in letrozole plus placebo treated patients. N.S., notsignificant. [file 13058_2022_1601_MOESM7_ESM.tif]

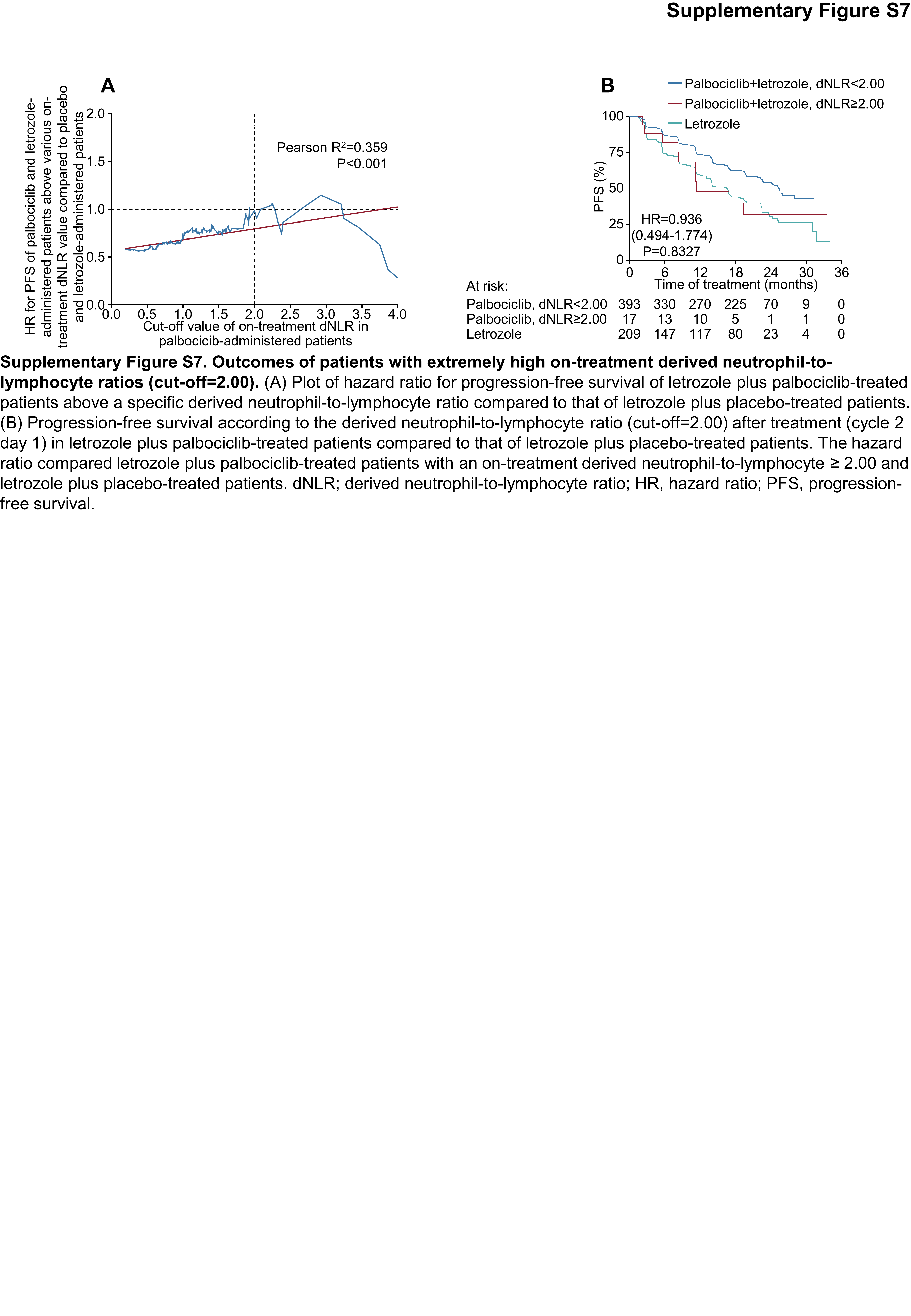

Supplement: Supplementary file 8 — Additional file 8. FigureS7: Outcome s of patients with extremely high on treatmentderived neutrophil to lymphocyte ratio s (cut off=2.00) (A) Plot of hazard ratio forprogression fre e survival of letrozole plus palbociclib treated patients above a specificderived neutrophil to lymphocyte ratio compared to that of letrozole plus placebo treatedpatients. (B) Progression free survival according to the derived neutrophil to lymphocyteratio (cut off=2.00) after treatment (cycle 2 day 1) in letrozole plus palbociclib treatedpatients compared to that of letrozole plus placebo treated patients. The h azard ratiocompar ed letrozole plus palbociclib treated p atients with an on treatment derived neutrophilto lymphocyte 2.00 and letrozole plus placebo treated patients. dNLR; derived neutrophilto lymphocyte ratio; HR, hazard ratio; PFS, progression free survival. [file 13058_2022_1601_MOESM8_ESM.tif]

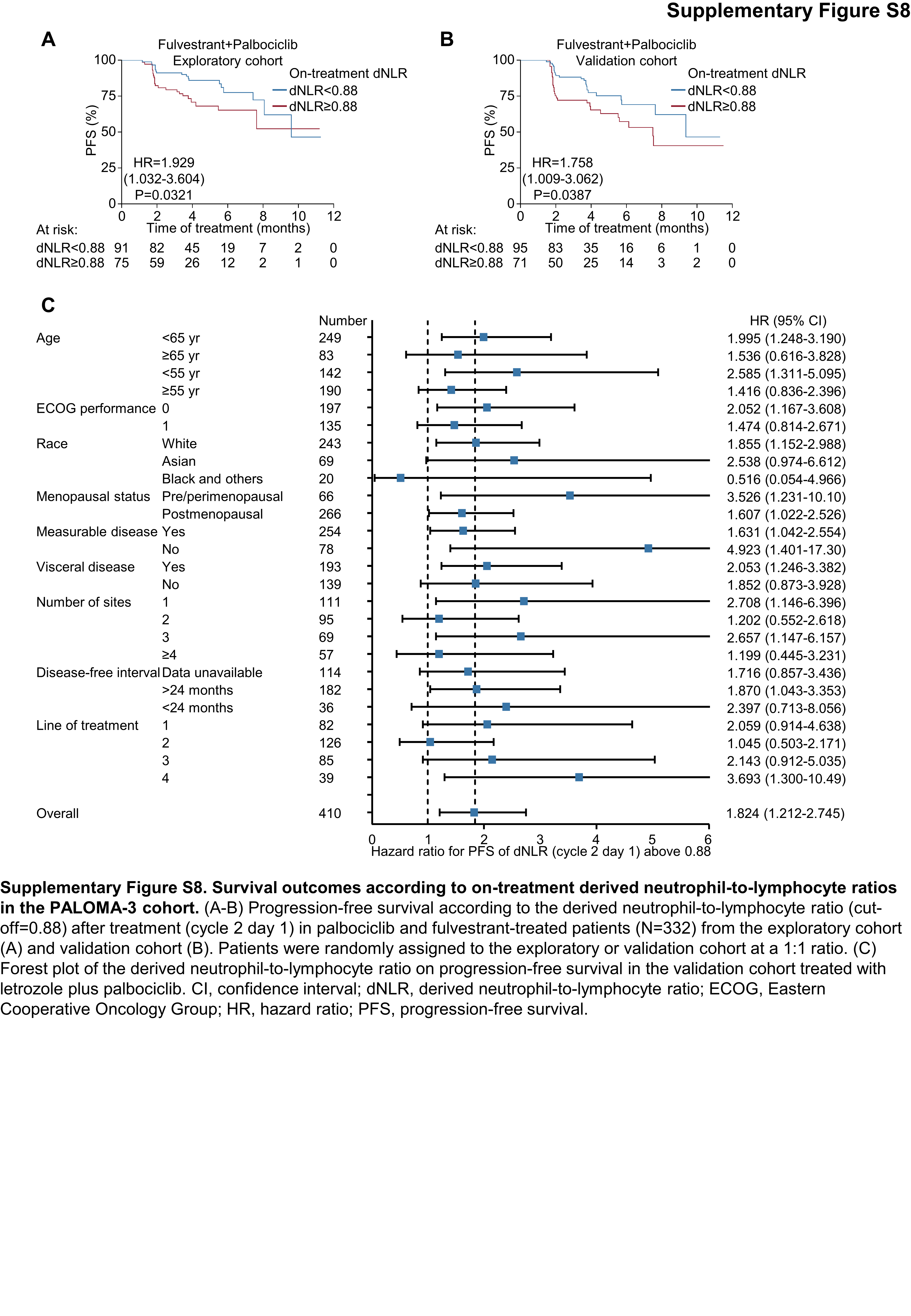

Supplement: Supplementary file 9 — Additional file 9. FigureS8: Survival outcome s according to on treatment derivedneutrophil to lymphocyte ratio s in the PALOMA 3 cohort. (A B) Progression freesurvival according to the derived neutrophil to lymphocyte ratio (cut off=0.88) aftertreatment (cycle 2 day 1) in palbociclib and fulvestrant treated patients (N=332) from theexploratory cohort (A) and validation cohort (B). Patients were randomly assigned to theexploratory or validation cohort at a 1:1 ratio. (C) Forest plot of the derived neutrophil tolymphocyte ratio on progression free survival in the validation cohort treated with letrozoleplus palbociclib. CI, confidence interval; dNLR, derived neutrophil to lymphocyte ratio;ECOG, Eastern Cooperative Oncology Group; HR, hazar d ratio; PFS, progression freesurvival. [file 13058_2022_1601_MOESM9_ESM.tif]

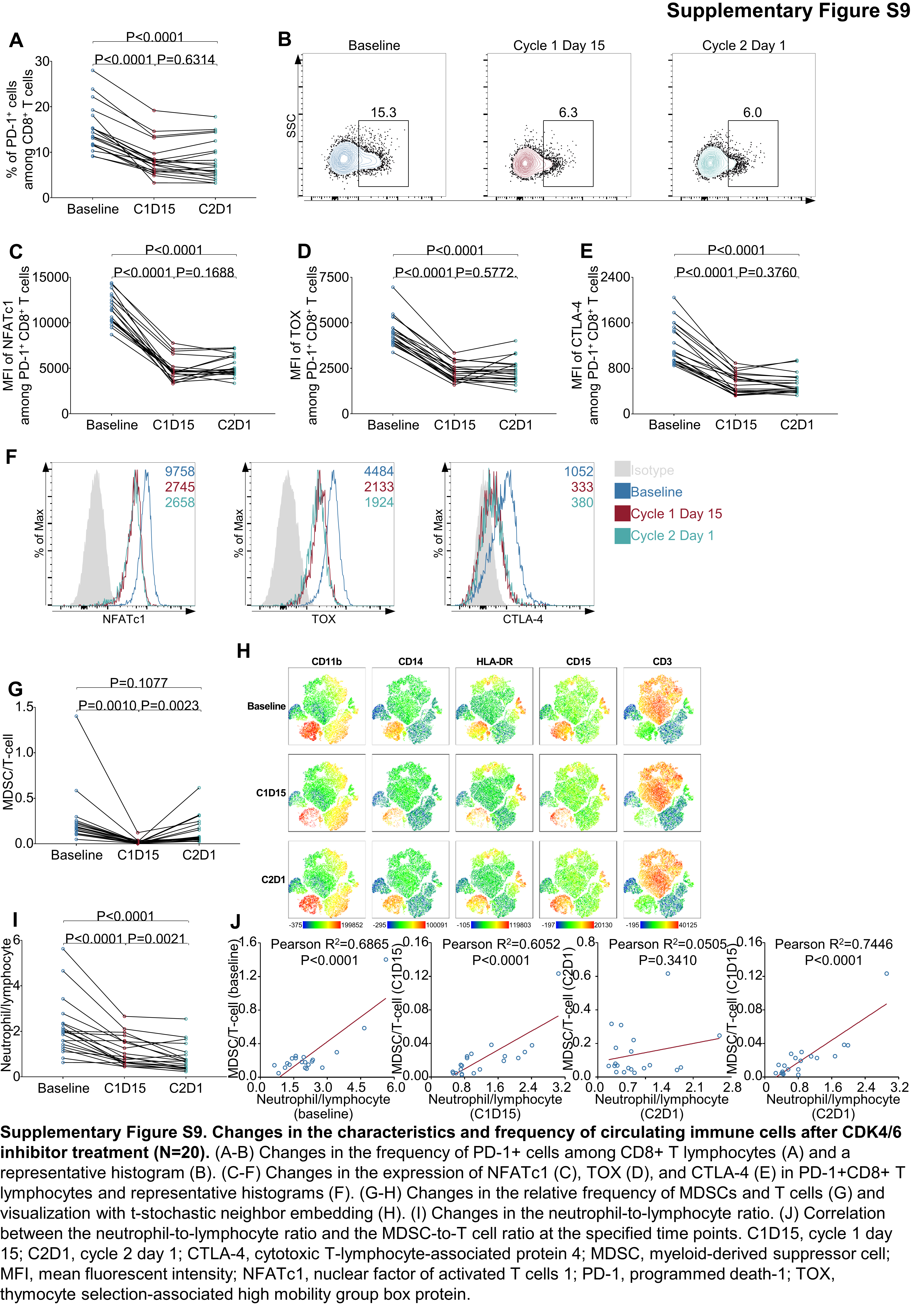

Supplement: Supplementary file 10 — Additional file 10. FigureS9: C hanges in the characteristics and frequency of circulatingimmune cells after CDK4/6 inhibitor treatment ( N = (A B) Changes in the frequency of PD1 cells among CD8 T lymphocytes (A) and a representative histogram (B) (C F)Changes in the expression of NFATc1 (C), TOX (D), and CTLA 4 (E) in PD 1 CD8 Tlymphocytes and representative histograms (F) (G H) Changes in the relative frequency ofMDSC s and T cells (G) and visualization with t stochastic neighbor embedding (H) (I)Changes in the neutrophil to lymphocyte ratio (J) Correlation between the neutrophil tolymphocyte ratio and the MDSC to T cell ratio at the specified time points. C1D15, cycle 1day 15; C2D1, cycle 2 day 1; CTLA 4, cytotoxic T lymphocyte associated protein 4; MDSC,myeloid derived suppressor cell; MFI, mean fluorescent intensity; NFATc1, nuclear factor ofactivated T cells 1; PD 1, programmed death 1; TOX, thymocyte selection associated highmo bility group box protein. [file 13058_2022_1601_MOESM10_ESM.tif]

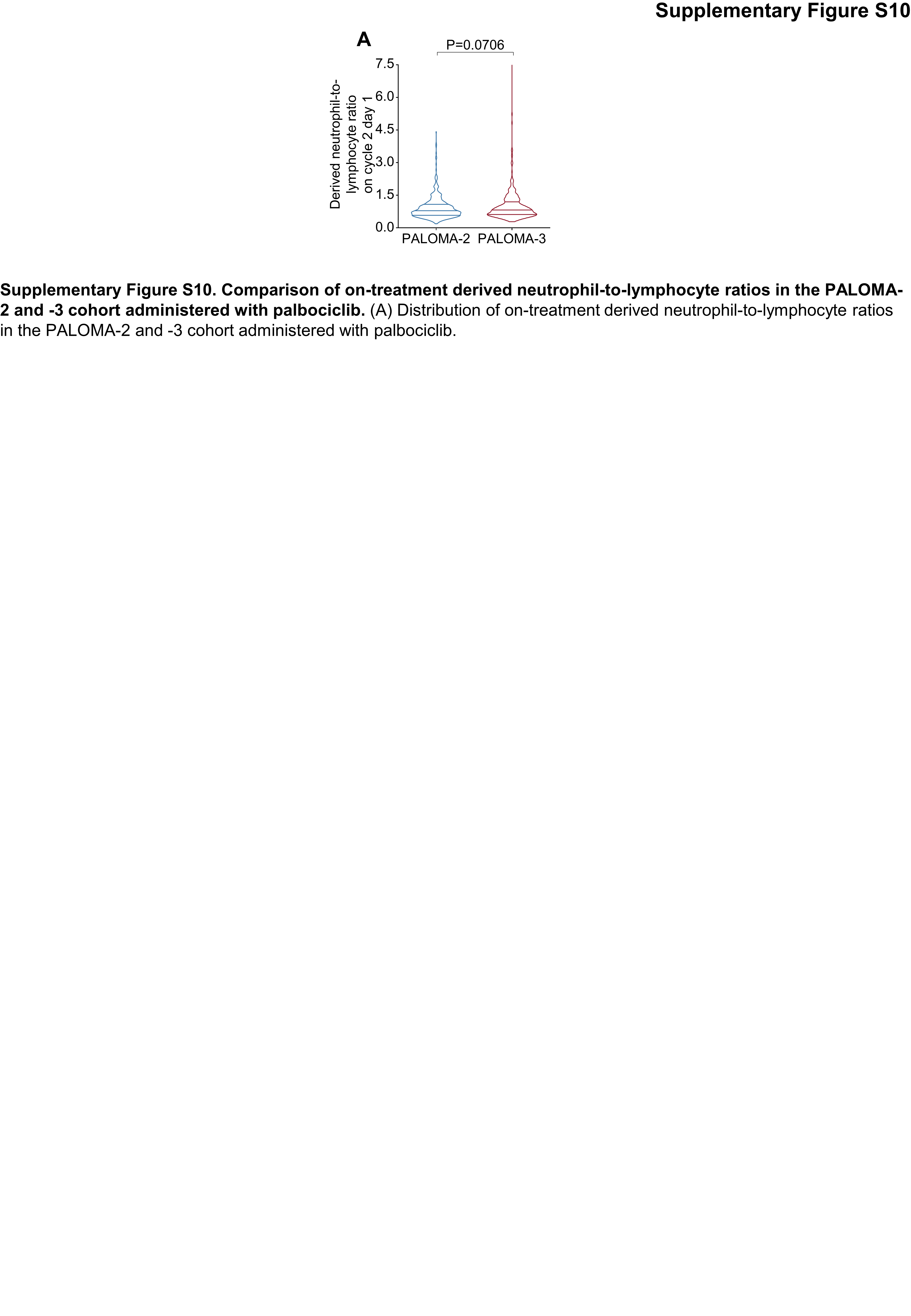

Supplement: Supplementary file 11 — Additional file 11. FigureS10: Supplementary Figure S10. Comparison of ontreatment derived neutrophil tolymphocyte ratios in the PALOMA 2 and 3 cohort administered with palbociclib. (A)Distribution of on treatment derived neutrophil to lymphocyte ratios in the PALOMA 2 and3 cohort administered with palbociclib. [file 13058_2022_1601_MOESM11_ESM.tif]
